# In the rivers: Multiple adaptive radiations of cyprinid fishes (*Labeobarbus*) in Ethiopian Highlands

**DOI:** 10.1038/s41598-020-64350-4

**Published:** 2020-04-28

**Authors:** Boris A. Levin, Evgeniy Simonov, Yury Y. Dgebuadze, Marina Levina, Alexander S. Golubtsov

**Affiliations:** 1 Papanin Institute of Biology of Inland Waters, Russian Academy of Sciences, Borok, Russia; 2 Cherepovets State University, Cherepovets, Russia; 3 Institute of Environmental and Agricultural Biology (X-BIO), University of Tyumen, Tyumen, Russia; 4 Tomsk State University, Tomsk, Russia; 5 Severtsov Institute of Ecology and Evolution, Russian Academy of Sciences, Moscow, Russia

**Keywords:** Evolution, Genetics, Zoology

## Abstract

Multiple repeated patterns of adaptive radiation were revealed in cyprinid fish inhabiting the compact geographic region of the Ethiopian Highlands. We found four independently evolved radiations in the evolutionary hexaploid (2n = 150) *Labeobarbus* lineage based on matrilineal relationships of >800 individuals. Each radiation displayed similar patterns of mouth phenotype diversification, and included ecomorphs/species of the generalized, lipped, scraping (one or two), and large-mouthed (one to three) types. All radiations were detected in geographically isolated rivers, and originated from different ancestral populations. This is the first documented case in which numerous parallel radiations of fishes occurred–via different ways–in a riverine environment. Some radiations are very recent and monophyletic, while others are older and include ecomorphs that originated in separate mini flocks and later combined into one. The diversification bursts among Ethiopian *Labeobarbus* were detected in the mid-upper reaches of rivers (1050-1550 m above sea level), which likely offer ecological opportunities that include diverse habitats yet poor fauna (i.e. lower competition and relaxed selection). This promising example of parallel evolution of adaptive radiation warrants further investigation.

## Introduction

The origin of biodiversity is one of the most intriguing questions in evolutionary biology and ecology. Along with the neutral divergence of geographically isolated lineages, adaptive radiation–the emergence of ecological and phenotypic diversity in rapidly diversifying lineages^1^–is considered to be the main mode that biodiversity is generated. Adaptive radiations usually result in biodiversity bursts in geographically restricted areas^2,3^. They often accompany the colonization of novel environments such as islands or lakes, or follow a massive species extinction when new ecological opportunities or niches become available^4–6^.

Fishes are the most diversified group of vertebrates. There are numerous examples of adaptive radiations occurring both in marine and freshwater environments^7–12^. Among freshwater fishes, the most impressive and species rich are lacustrine radiations seen in various systematic groups (e.g. cichlids, sticklebacks, salmonids, cyprinids, catfishes and many others; see^8,12–15^ for reviews.

Although examples of adaptive radiation in riverine fish are not particularly frequent, they have substantially increased during the last decades. Such evolutionary phenomena have been revealed by molecular data in the African mormyrids, mochokid catfishes and cyprinids^16–20^, as well as in the African and South American cichlids^21–27^. For example, cichlids of the genus *Crenicichla* Heckel 1840 from Paraná and Uruguay Rivers demonstrate bright morpho-ecological diversification and trophic resource partitioning. In particular, five co-occurring ecomorphs diverged in trophic ecology, and are of recent origin based on genetic data^27^. The cyprinids of the *Labeobarbus* Rüppell 1835 from the Genale River, East Africa, constitute a sympatric assemblage of six ecomorphs, five of which are divergent in trophic ecology. All riverine forms of the *Labeobarbus* from the Genale River have intra-basin origins^20^. Apart these cases, there is evidence of several other putative riverine adaptive radiations in the African and Asian cyprinids^28–32^, though they have yet to be confirmed with genetic data.

In the pioneering work on South African cichlids and catfishes, Joyce *et al*.^21^ and Day *et al*.^19^ suggested that the riverine flocks are remnants of radiations that emerged in a paleolake. This suggestion is corroborated by the finding of the Lake Tanganyika endemics beyond the lake basin^33^. While some riverine radiations clearly have lacustrine origins, it is likely that other riverine radiations originate from sources other than lakes. Hence, we aimed to test whether the same environmental factors that trigger lacustrine radiation can also act in rivers.

In general, the main prerequisites for adaptive radiation are: (1) environmental stability for evolutionarily significant periods (starting from thousands of years ago); and (2) physical isolation from the river drainages harboring diverse fish faunas^15,20,31^. The latter serves a dual purpose: first, it provides the ecological opportunities for niche divergence at the initial stages of radiation, and later it prevents the extinction of diverged forms caused by competition with highly specialized species from other systematic groups. These processes can operate in specific and rather rare parts of the riverine network. For example, the segment of the Genale River that harbours the radiating assemblage of distinct forms of the *Labeobarbus* is situated in the apparently old canyon, isolated by the Baratieri Falls from the lower reaches of the river system and characterized by the depauperate fish fauna^20^.

If the fish radiations in rivers are similar to the evolutionary phenomena experienced in lakes, they should display similar evolutionary patterns. It is well known that adaptive radiations are often repeatable in different organisms as exemplified from spiders^34^, fish^35^, and lizards^36^. Moreover, lacustrine radiations in many fish groups are usually parallel or convergent. Clear examples can be found among cichlids^37,38^, sticklebacks^39–41^, Arctic charrs^42,43^ and whitefishes^44–46^. As for riverine radiations, to the best of our knowledge, the only genetically proven example is the parallel trophic diversifications of the cichlids of the genus *Crenicichla* in two South American rivers^27^.

The large African barbs of the genus *Labeobarbus* belong to the African Torini, a lineage of evolutionary hexaploids (2n = 150)^47–49^ that originated in the Middle East via hybridization of the evolutionary tetraploids (maternal *Tor* Gray 1834 lineage) and diploids (paternal *Cyprinion* Heckel 1843 lineage), which then dispersed throughout the African continent^50^. This lineage is distributed in ten African ichthyofaunal provinces as defined by Snoeks *et al*.^51^ and includes approximately 125 species^52^. Its origin supposedly dates back to *ca*. 7.6 Mya BP^53^, which corresponds to the earliest fossil records of *Labeobarbus* of late-Miocene age at the middle reaches of the Awash River in the Ethiopian Rift Valley^54^. On one hand, the origin of *Labeobarbus* via ancient hybridization, as well as their high level of ploidy, inhibit the use of nuclear markers in the analyses of their phylogeny. On the other hand, their complex evolutionary history promotes the extreme variability in mouth phenotype and body shape in this group^20,52^. The large African barbs exhibit multiple cases of parallel morphological and ecological divergence in the feeding-related characters at both intra- and interspecific levels (e.g.^15,20,52,55–70^).

The well-known example of the morphological and ecological divergence of *Labeobarbus* in Lake Tana, northwestern Ethiopia, is usually considered as one of the two largest lacustrine species flocks in cyprinids^71^. However, monophyly of the Tana flock, which includes up to 16 forms/species, is not yet substantiated^72^. The same is true for the second largest flock of cyprinids from Lake Lanao, Philippines^73^, which became extinct before most of its forms were studied genetically^74^. Recently, an assemblage of morphologically and ecologically distinct *Labeobarbus* forms from the Genale River in the Indian Ocean catchment, southeastern Ethiopia, was tested for monophyly^20^. It was shown that the Genale assemblage appears to have originated through a combination of allopatric and sympatric events, but all diversification took place within the solitary large river system in southeastern Ethiopia.

**Figure 1:**
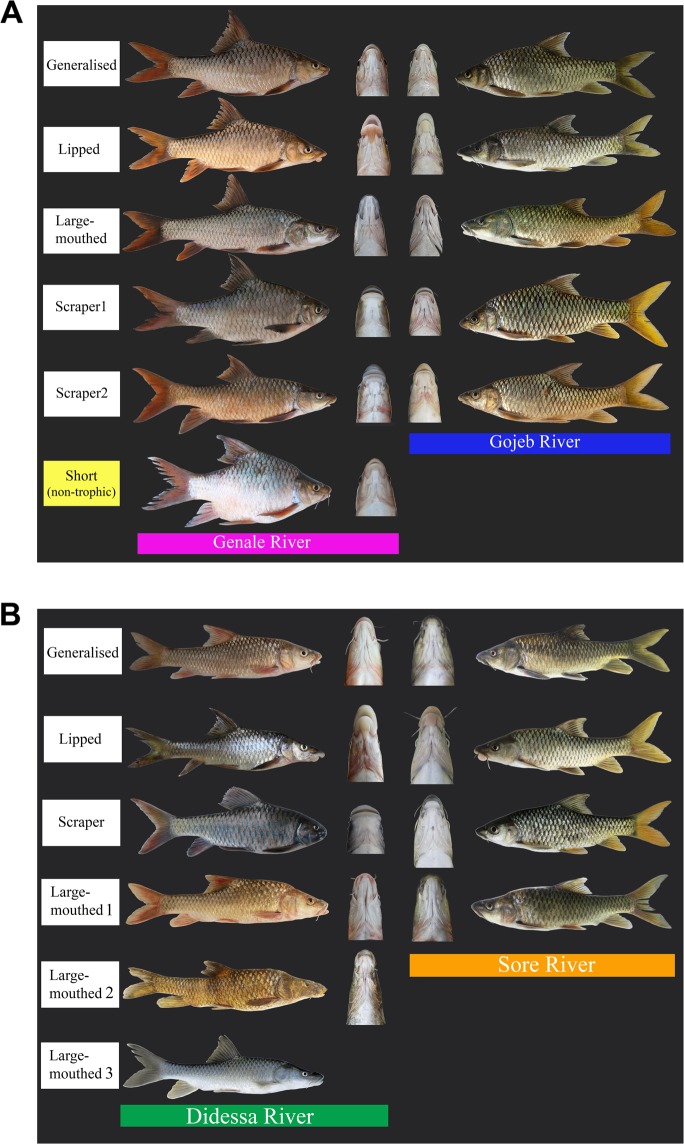
(**A**) Appearance of ecological forms of *Labeobarbus* in different rivers of the Ethiopian Highlands. Left: the Genale River (Juba-Wabe-Shebelle drainage), and right: the Gojeb River (Omo-Turkana drainage). (**B**) Appearance of ecological forms of *Labeobarbus* in different rivers of the Ethiopian Highlands. Left: the Didessa River in Blue Nile basin, and right: the Sore River in White Nile basin.

Western Ethiopia, including the Ethiopian Rift valley and Lake Tana basin, is populated by the *Labeobarbus intermedius* Rüppel 1835 supercomplex that is a sister group of the *L. gananensis* (Vinciguerra 1895) /*L. jubae* (Banister 1984) complex occurring in southeastern Ethiopia^53^. Additionally, morphological diversification in the feeding-related characters was reported from the three river (Blue Nile, White Nile and Omo River) drainages within the range of the *Labeobarbus intermedius* supercomplex^15,61^. Similar to the Genale River, in all three western basins the assemblages of sympatric forms include i) generalised (or intermediate) phenotype, ii) lipped phenotype, iii) scraping or chisel-mouthed phenotype(s), and iv) large-mouthed phenotype(s) (Table 1, Fig. 1).

**Table 1:** Riverine assemblages of ecological forms of *Labeobarbus* spp. in isolated riverine basins in the Ethiopian Highlands.

River and basin	Mouth phenotypes detected	Source
1. Genale River, Juba-Wabe-Shebelle basin, Indian Ocean drainage (loc. 1 on Fig. 2)	- generalised - lipped - scraping (two forms) - large-mouthed	Dimmick *et al*., 2001^63^; Golubtsov, 1993^75^; Levin *et al*., 2019^20^; Mina *et al*., 1998^61^
2. Gojeb River, Omo-Turkana basin (loc. 37)	- generalised - lipped - scraping (two forms) - large-mouthed	Golubtsov, 2010^15^; our observation
3. Didessa River, Blue Nile basin, Atlantic Ocean drainage (loc. 21-22)	- generalised - lipped - scraping (*L. beso*) - large-mouthed (*L. zaphiri* and two undescribed forms)	Golubtsov, 2010^15^; Mina *et al*., 1998^61^; our observation
4. Sore River, White Nile basin, Atlantic Ocean drainage (loc. 25-26)	- generalised - lipped - scraping - large-mouthed	Golubtsov 2010^15^; our observation

**Figure 2:**
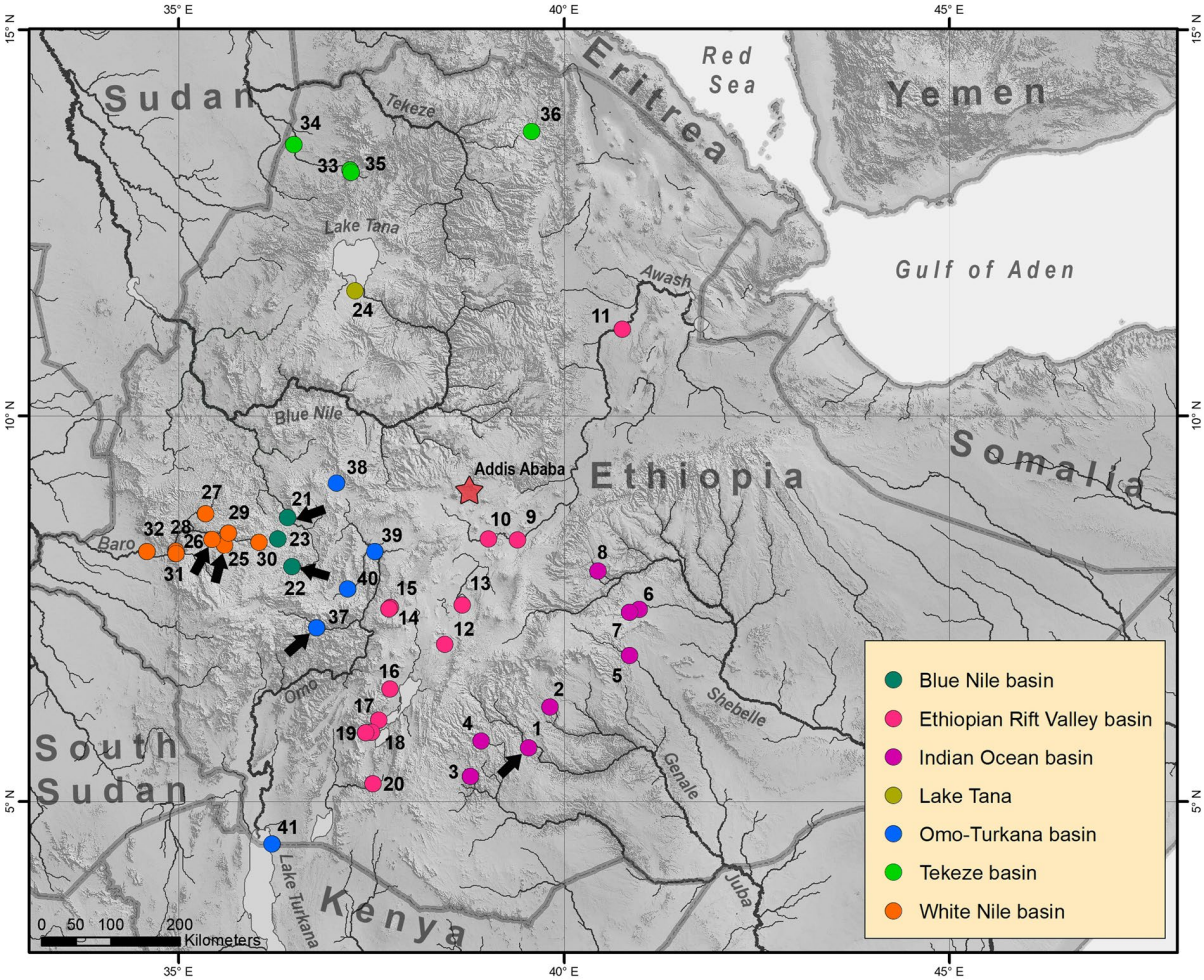
Map of sampling sites. Black arrows point the localities, where riverine assemblages of ecological forms of *Labeobarbus* recorded: 1–Genale River, 21-22–Didessa River, 25-26–Sore River, and 37–Gojeb River. Map was created in ArcGIS 10.2 software (www.esri.com).

The main problem we addressed in this study was the origin of the geographically close *Labeobarbus* assemblages from the rivers of western Ethiopia. We used mitochondrial cytochrome *b* (*cytb*) gene sequences in the barbs obtained from more than 40 localities across Ethiopia (Fig. 2) to estimate: (1) the independent origin of *Labeobarbus* assemblages from the White Nile, Blue Nile and Omo River drainages, (2) monophyly of each of these assemblages, and (3) genetic distinctiveness of the morphologically diverged forms within the assemblages.

## Results

### Phylogeny of *Labeobarbus* in the Ethiopian Highlands

**Figure 3:**
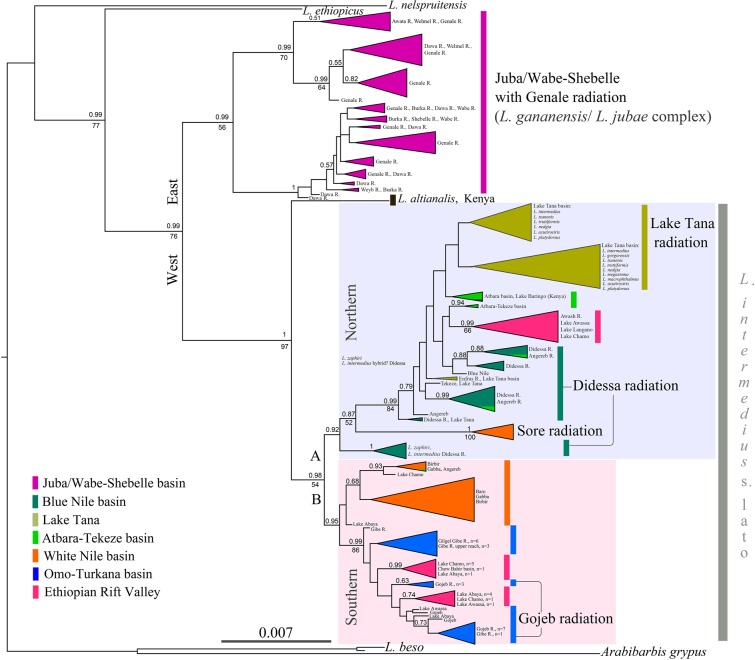
Bayesian inference majority-rule consensus tree of relationships among the Ethiopian *Labeobarbus* from all main drainages including all unique haplotypes based on *cytb* sequences. Bayesian posterior probabilities (above) from BI analysis and bootstrap values from ML analysis (beneath) above 0.5/50 are shown. Scale bar and branch lengths are given in expected substitutions per site. The nodes were collapsed to a triangle, with the horizontal depth indicating the level of divergence within the node. The phylogenetic tree was visualized using FigTree v.1.4.4^106^.

Both Bayesian inference (BI) and maximum likelihood (ML) analyses supported the division of Ethiopian *Labeobarbus* into two clades representing the eastern (*L. gananensis/L. jubae* complex) and western (*L. intermedius* supercomplex) parts of the Ethiopian Highlands, with *L. ethiopicus* (Zolezzi 1939) as an outgroup (Fig. 3). The western clade also includes *L. altianalis* (Boulenger 1900) from the Lake Victoria basin in Kenya, which is a sister lineage to all *Labeobarbus* in this clade. Ethiopian *Labeobarbus* in the western clade are further subdivided into A) northern, and B) southern lineages. Lineage A includes populations from the Blue Nile basin with Lake Tana, the Atbara-Tekeze basin (the Nile), the mainly northern localities from the Ethiopian Rift Valley (the Awash system, Lakes Langano, Awassa and several representatives from Lakes Abaya and Chamo). Surprisingly, this lineage also includes one population from the White Nile basin (Sore River). Two riverine (the Didessa and Sore) and one lacustrine (Lake Tana) radiations were nested within northern lineage. The southern lineage is comprised of populations from the White Nile basin, with the exception of the Sore population, and populations from the Omo-Turkana and the southern part of the Ethiopian Rift Valley (Lakes Awassa, Abaya, Chamo, and Chew Bahir basin) (Fig. 3). This group includes *Labeobarbus*’ diversification detected in the Gojeb River, a tributary of the Gibe River (Omo-Turkana system). These northern and southern lineages correspond to lineages A and B suggested by Beshera & Harris^72^, but include more basins and localities due to our wider geographic coverage. The remaining *L. ethiopicus* and *L. beso* (Rüppell 1835) that are endemic to Ethiopia but placed in an outgroup position to all other Ethiopian *Labeobarbus*.

Although the intragroup divergence was often weakly resolved or not resolved, the monophyly was supported by the Bayes factor test for the Gojeb (log BF = 7.82) and Sore (log BF = 8.10) assemblages, confirming their species flock status. However, there was no such support for the Didessa (log BF = 51.33 in favor of unconstrained topology) and Genale (log BF = 394.6) assemblages.

### *Cytb* haplotype network/phylogeography

**Figure 4:**
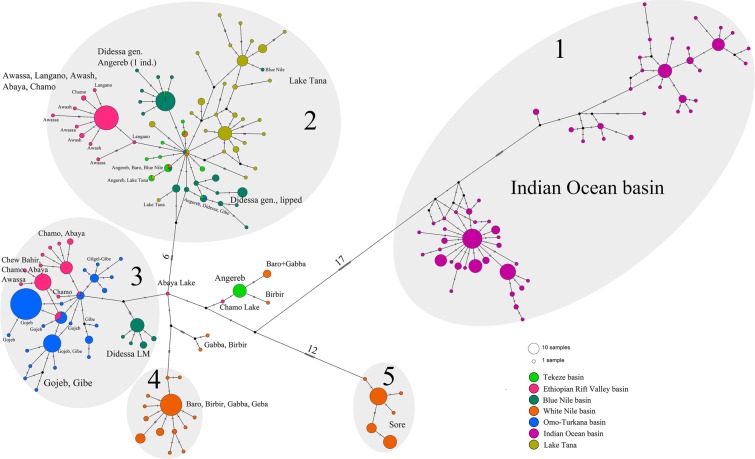
Median-joining haplotype network of the Ethiopian *Labeobarbus* from all main basins, constructed based on 769 *cytb* sequences. Haplogroup 1 corresponds to the *L. gananensis*/*L. jubae* complex, while haplogroups 2–5 join *L. intermedius* s. lato. Black dots represent hypothetical intermediate haplotypes. Haplotype network was built with PopART 1.7 software^108^.

Among the 769 individuals of Ethiopian *Labeobarbus* sampled, 185 haplotypes were detected. The haplotype network is complex (Fig. 4), composed of five main haplogroups with a central haplotype represented by an individual from the Ethiopian Rift Valley (Fig. 4). Haplogroup (1) includes the most diversified labeobarbs of the *L. gananensis*/*L. jubae* complex from the Genale River and other tributaries of the ancient Juba-Wabe-Shebelle (JWS) drainage, with 24 mutational steps to the nearest haplotype from the Chamo Lake in the southern Ethiopian Rift Valley. Haplogroup (2) is also diverse and includes the lacustrine radiation of *Labeobarbus* from Lake Tana, along with populations from the Blue Nile (including the Didessa *Labeobarbus*, represented by two divergent compact haplogroups), the Nile (Tekeze basin), and populations mainly from the northern part of the Ethiopian Rift Valley. Haplogroup (3) comprises the riverine radiation of the Gojeb barbs within the Gibe River, as well as populations from the southern part of the Ethiopian Rift Valley including Lake Turkana. The portion of the Didessa radiation that is mainly composed of forms with the large-mouthed phenotype is very close to the Omo-Turkana haplogroup. Haplogroups (4) and (5) are much more compact and both represent the tributaries of the White Nile basin. While haplogroup (4) includes most samples from the Baro River and its tributaries, haplogroup (5) includes only the population from the Sore River, a radiation of *Labeobarbus* reported in the current study. These haplogroups were separated by 23 mutation steps and joined via a central haplotype.

**Figure 5:**
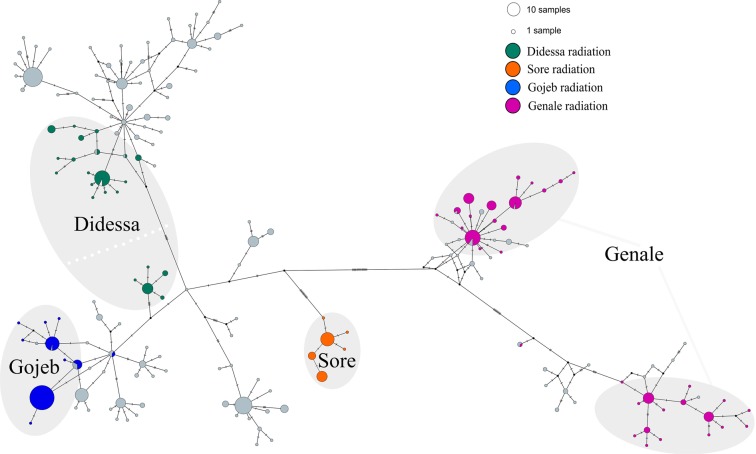
Median-joining haplotype network of the Ethiopian *Labeobarbus* from all main basins, constructed based on 769 *cytb* sequences. Only riverine radiations (Genale, Didessa, Gojeb, and Sore) are colored. Black dots represent hypothetical intermediate haplotypes. Haplotype network was built with PopART 1.7 software^108^.

For ease of visualization, Fig. 5 highlights only the riverine adaptive radiations. Based on both the *cytb* phylogenetic trees and haplotype network, all four radiations appear to have evolved independently. Each originated within its riverine basin, except for the Didessa radiation (see below):

  (I). The Genale radiation occurred within the Juba and Wabe-Shebelle basin (Indian Ocean drainage); it displays the deepest divergence and highest haplotype diversity (Table 2), which is consistent with previous results from a recent study^20^.

**Table 2:** Genetic variation within riverine radiations based on *cytb* sequences.

Basin	*n*	*H*	*h* ± SD	*π* ± SD	*K*
Genale	151	34	0.91 ± 0.011	0.0088 ± 0.00042	9.1
Gojeb	122	10	0.52 ± 0.046	0.0009 ± 0.00010	1.0
Didessa	60	12	0.80 ± 0.037	0.0058 ± 0.00030	6.1
Sore	49	5	0.65 ± 0.043	0.0015 ± 0.00008	1.5

*n–*sample size; *H–*number of haplotypes; *h–*haplotype diversity; *π–*nucleotide diversity (per site); *K–*average number of nucleotide substitutions; SD–standard deviation.
 (II). The Gojeb radiation occurred in the Gojeb-Gibe (Omo-Turkana basin). Some Gojeb individuals share haplotypes with or are genetically similar to a population from the southern part of the Ethiopian Rift Valley.
(III).  The Sore radiation was realized within the smallest haplogroup specific for the Sore River. Although the Sore River belongs to the Baro riverine system (White Nile), its haplotypes are distant to any others within the same basin (17 mutations to the closest haplotype). Here the Sore barbs can be considered as its own evolutionary lineage within White Nile basin. It is likely that this lineage is old (it has early divergence in the phylogenetic tree - Fig. 3), but the radiation is recent, as it has the lowest genetic and morphological diversity (unpubl. data) compared to the other three riverine radiations.
(IV).  The origin of the Didessa radiation is more complicated than that of the other three radiations, which occurred within their respective basins. The scraper form *L. beso*, which is phenotypically analogous to the scraping forms in other radiations, is distant to any of the other Ethiopian barbs and branched significantly earlier (in outgroup position in Fig. 3). Most of the haplotypes of the three large-mouthed forms belong to the Omo-Turkana haplogroup, although they were separated from the radiation detected in the Omo-Turkana by 6 mutational steps. Only few individuals shared haplotypes with the co-occurring generalized form from the Didessa, most likely due to introgression, considering the large genetic distance between these two (Figs. 3–4, S3). The group of the Didessa large-mouthed forms within Omo-Turkana haplogroup does not share haplotypes with any other forms, and was also distant to other sympatric radiations like Gojeb and Sore. This most likely represents a remnant of the past flocks like the scraper *L. beso*. Therefore, the Didessa radiation is not of sympatric origin (except for the generalised-lipped phenotypes; see below for discussion). The generalised, scraper and large-mouthed forms originated from different ancestors. Interestingly, despite this complex history, the forms in the Didessa are very similar to the forms from other basins in which sympatric origin was detected. This raises the question as to whether the riverine environment strongly and predictably shapes the adaptive landscape of the *Labeobarbus*?

### Genetic diversity within adaptive radiations and intra-radiation relationships

The highest haplotype diversity was detected in the Genale (*h* = 0.91) and Didessa (*h* = 0.80) radiations, which are non-monophyletic in origin (Levin *et al*.^20^ and this study), compared to the Sore (*h* = 0.65) and Gojeb (*h* = 0.52), which had much lower nucleotide diversity and average number of nucleotide substitutions (Table 2).

The genetic differentiation between sympatric forms was revealed within the Genale and Didessa riverine radiations in F_ST_ values (Table S4). No subdivision or incomplete lineage sorting between sympatric phenotypes was detected within the Gojeb and Sore riverine radiations. However, some phenotypes in these rivers had haplotypes that were divergent from sympatrically occurring phenotypes. In particular, the large-mouthed phenotype differed from the generalized, lipped and scraper1 in the Gojeb River, the while in the Sore River the scraper phenotype differed from the generalized form (Table S4).

## Discussion

The most important result of our study is the evidence for repeatedly evolved similar patterns of ecological diversification (namely in mouth phenotypes) in a compact geographic region. We found that cyprinid fish *Labeobarbus* exhibit parallel adaptive radiation in the four rivers in the Ethiopian Highlands that belong to independent riverine drainages. Although this phenomenon is commonly observed in lake-dwelling fishes (e.g.^1,38,42,43,46,76,77^), it is extremely rare in river-dwelling fish assemblages. Thus far, only one case of repeated patterns composed of two riverine adaptive radiations has been documented within the South-American cichlids of the genus *Crenicichla* from the Paraná and Uruguay Rivers^25,27^. The four riverine radiations of Ethiopian *Labeobarbus* originated from different ancestral populations. Moreover, our mitochondrial data suggests that the repeated patterns of phenotypic diversification also have different origins, which we discuss below.

Two of the four assemblages–the Gojeb (Omo-Turkana basin) and Sore (White Nile)–are monophyletic in origin without complete haplotype sorting between sympatric forms. This finding can most likely be explained by their recent origin. Nevertheless, some sympatric phenotypes in these radiations differed significantly in haplotype frequency, which may indicate reproductive isolation (Figures S1-S3; Table S4). These two can be considered as species flocks (sensu^78^) and their sympatric origin is a plausible hypothesis. Both the Gojeb and Sore species flocks have significantly lower haplotype and nucleotide diversity compared to the Genale and Didessa assemblages. The Sore radiation differs from the Gojeb in that it has fewer forms (4 vs. 5). Moreover, the low F_ST_ values suggest a younger origin or slower diversification rate of the Sore radiation compared to the Gojeb, which is supported by our morphological data (unpublished) that also revealed shallower phenotypic divergence among the sympatric Sore forms. Notably, the Sore lineage showed early branching in the phylogenetic tree, which would suggest that this relatively old lineage has only recently started to diversify.

The origin of the other two radiations (Genale and Didessa) is more complex. Our data indicate that their assemblages are the result of a combination of sympatric and allopatric speciation with secondary contact due to geological events during ancient periods. A recent study highlighted that the diversification of the *Labeobarbus* from the Genale River is a result of three mini species flocks that originated in different channels of an ancient riverine net with a dynamic geological history^20^. Two of the specialized Genale forms, large-mouthed and one of the scrapers, originated in two geographically distant parts of the riverine basin and later colonized/migrated to the Genale River where they combined with local forms (generalised, lipped, and short form)^20^. Therefore, the Genale adaptive radiation is of mixed origin fueled by both sympatric and allopatric speciation (see Levin *et al*.^20^ for details). For such a diversification that results from the combination of products from different mini flocks of closely related lineages, we have suggested a term multiflock^20^.

Similar to the Genale, diversity of the Didessa *Labeobarbus* assemblage is a result of the combination of sympatric and allopatric speciation (generalised and lipped forms) during different geological time. The scraper form of the Didessa River, *L. beso*, is a morphologically highly specialized algae scraper^67^ with more ancient origin than any *Labeobarbus* in the Ethiopian Highlands based on its phylogenetic position^66,68^. It is possible that *L. beso* is a relic remnant of a former (extinct) species flock that existed before the recent lineages of the *Labeobarbus* colonized the Ethiopian Highlands. This is plausible in light of the fact that the Ethiopian Highlands is a tectonically active territory with a recent Plio-Pleistocene volcanism^79–81^. An ancient species flock hypothesis was recently proposed to explain the origin of diversity within another lineage of algae scrapers of the genus *Capoeta* Valenciennes 1842 in the Armenian Highland^82^.

The *Labeobarbus* earliest fossil records were found in the Ethiopian Rift Valley and dated back to the late-Miocene^54^. The Ethiopian Highlands are a volcanic massif of flood and shield volcano basalts 0.5–3.0 km thick that form spectacular trap topography (1500–4500 m) flanking the Main Ethiopian Rift^81^. The geological history of the Ethiopian Highlands was very dynamic and rich in volcanic episodes; four main periods have been detected from Oligocene to Pleistocene time^81^. The volcanic activity has been severe enough to deleteriously affect the biota and cause major disruptions in ecosystems. This could likely explain i) the rare representatives of older branches among Ethiopian *Labeobarbus* (*L. beso* and *L. ethiopicus*), and ii) the young, Pleistocene origin of the majority of *Labeobarbus* species and populations^53^.

We uncovered three large-mouthed phenotypes in the Didessa River with no intra-divergence in mtDNA; these are genetically distant from sympatric generalised and lipped phenotypes, as well as any individuals from the Blue Nile-Lake Tana system. In a haplotype network, the Didessa large-mouthed forms had an intermediate position between populations from the Gojeb (Omo-Turkana) and the central haplotype (Ethiopian Rift Valley). We hypothesize that the large-mouthed phenotypes have diverged within an ancient flock that is undetected now. Accordingly, co-existence of sympatric ecomorphs in the Didessa would be the result of both sympatric speciation and ancient secondary contacts. Of course, diversification of the three closely-related large-mouthed (piscivorous) phenotypes in the riverine environment is an intriguing phenomenon for cyprinids and should be studied further. We cannot exclude the scenario that these phenotypes (and possibly others as well) diversified upon secondary contact and as a result of genetic admixture. This scenario was successfully tested to explain the origin of functional novelties in some cichlids^83^, and also the contemporary ecological speciation of threespined stickleback in Lake Constance^84^. The ancient hybridization between divergent lineages of cichlids was also reasonably hypothesized to fuel mega-radiations in African Great Lakes^85–87^.

To summarize, the very similar patterns of adaptive radiations in riverine drainages of the Ethiopian Highlands were achieved via different processes:

- Sympatric ecological speciation, when all members of the adaptive radiation are of monophyletic origin, i.e. represent species flock and sympatric speciation (Gojeb and Sore).
- Secondary contact of closely-related but reproductively isolated specialized phenotypes, which originated in different parts of the same riverine basin. This is essentially established for the Genale River multiflock^20^.
- Secondary contact of closely-related but genetically isolated pools of phenotypes, which could initiate ecological speciation upon hybridisation (this scenario should be tested further using more comprehensive genomic approaches). Indeed, a similar scenario was recently suggested for *Labeobarbus* from the Lower Congo^69^.

Parallel origin of the very similar mouth polymorphism of *Labeobarbus* spp. suggests that these phenotypes are determined by several factors. For example, such propensity to produce different mouth phenotypes is apparently explained by a complex hybrid genome. The *Labeobarbus* is an evolutionary hexaploid fish of allopolyploid origin^47,48,50^. Its maternal lineage was from the tetraploid *Arabibarbus* Borkenhagen 2014 *(=Tor)* lineage^50^, distributed in the Middle East. Mainly representatives of the *Arabibarbus* have a generalised mouth with moderately developed lips, while few ones possess hypertrophied *rubber lips*^88,89^. The paternal lineage of the *Labeobarbus* is diploid *Cyprinion*, distributed in South Asia and the Middle East. Most *Cyprinion* species are specialized algae scrapers with a well-developed horny sheath on the lower jaw^89^. Hereby, a mouth polymorphism of *Labeobarbus* is apparently based on pre-existing genetic templates, a legacy of ancestors, and realized numerously as replicated pattern in plethora of isolated lineages distributed throughout Africa. Meanwhile, the novel phenotypes such as scraper2 in the Ethiopian Highlands, papillated mouth phenotype in West Africa, and numerous diversified large-mouthed phenotypes (e.g.^59^) are likely the results of the new genomic combinations during further evolution.

Such a genomic prerequisite is highly important but not an exclusive explanation for the phenotypic diversification of Ethiopian *Labeobarbu*s. Adaptive radiation is often linked to colonization of a new environment that is somehow isolated from further colonists like islands or lakes, which provide new ecological opportunities^1,90^. The significant elevation of the Ethiopian Highlands formed due to several episodes of volcanisms with one of the largest explosive volcanic events in Earth’s history^81^, which probably had deleterious effects on local biota. The last major episode of volcanism in the Ethiopian Highlands was in the Pliocene-Quaternary period, however it was very recent in certain regions (33 kya Blue Nile basaltic blockade formed Tis-Isat waterfall^81^). Indeed, the oldest fossils of *Labeobarbus* (late Miocene) were found in the Northern Ethiopian Rift Valley^54^ but the main diversification among the Ethiopian *Labeobarbus* is of more recent origin (Pleistocene) in Africa^53^. Based on published phylogenetic trees^53^ (and our study), the ancestor lineage that colonized the Ethiopian Highlands was from the Kenyan water bodies, like *L. altianalis* widely distributed in East African Rift from the northern part of Lake Tanganyika northward to Lakes Victoria and Kyoga.

The *Labeobarbus* is distributed along the rivers of the Ethiopian Highlands that vary gradually in several ways. For example, the elevation gradient from our sampled locations ranged from 175 m to ca. 2000 m above sea level. Interestingly, the diversification bursts were only detected in the mid-upper segments of the rivers varying between 1050 (Gojeb) and 1550 m (Sore) above sea level. This altitude effect can be attributed to a combination of two factors. First, the fauna in the mid-upper reaches is commonly poorer compared to that in the lower reaches (e.g.^15,20^), where more diversified fauna–including highly specialized competitors–can be found. Hence, natural selection in the fauna-poor upper reaches might be relaxed due to lowered competition. Relaxed selection can help to increase morphological and ecological variability, leading to subsequent diversification. The second factor that likely contributes to the increased diversification in the middle reaches is the increased availability of ecological niches (i.e. more diverse ecotopes/habitats in this section offer more ecological opportunities). We suggest that continued diversification bursts among *Labeobarbus* are possible in both Ethiopian Highlands and elsewhere in its range.

## Material and Methods

### Ethic statement

Fish were sacrificed by state-of-the-art humane killing using anesthetic overdose (American Veterinary Medical Association). The experiments were carried out in accordance with the rules of the Papanin Institute of Biology of Inland Waters (IBIW), Russian Academy of Sciences and approved by IBIW’s Ethics Committee.

### Sample collection

Fishes were sampled in the water bodies of Ethiopia during 2008-2018 in the framework of the Joint Ethio‐Russian Biological Expedition (JERBE), with sampling permission from the appropriate authorities. Tissue samples were collected from 664 specimens of *Labeobarbus gananensis, L. jubae* and *L*. cf. *intermedius* from 41 localities, including four putative riverine radiations (Fig. 2, Table S1) from all main drainages of Ethiopia: i) Indian Ocean catchment (Juba and Wabe-Shebelle drainage), ii) Atlantic Ocean catchment (Blue Nile, White Nile and Nile), iii) enclosed Omo-Turkana, and iv) Ethiopian Rift Valley. DNA vouchers were deposited to the Severtsov Institute of Ecology and Evolution Russian Academy of Sciences and Papanin Institute of Biology of Inland Waters Russian Academy of Sciences. Map of sampling sites (Fig. 2) was created in ArcGIS 10.2 software (www.esri.com).

### DNA extraction, PCR amplification and sequencing

DNA was extracted from a small piece of the fin or muscle using a standard salt method^91^ or a BioSprint 15 kit for tissue and blood (Qiagen). A 1037 bp fragment of the mtDNA *cytb* gene was amplified by PCR using the following primers: GluDg: 5′-TGACTTGAARAACCAYCGTTG-3′^92^ and H16460: 5′-CGAYCTTCGGATTAACAAGACCG-3′^93^. PCRs were carried out in 25–50 μl reactions [1× buffer, 1.5 μM MgCl2, 0.5 mM of each primer, 0.2 μM of each dNTP, 1 μl template DNA, and 1U Taq polymerase (Eurogene, Moscow)] under the following conditions: 94 °C (2 min), 30 cycles at 94 °C (45 s), 48 °C (1 min), 72 °C (90 s), and a final extension at 72 °C (5 min). PCR products were visualized on 1.5% agarose gels and later purified via ethanol/3 M ammonium acetate precipitation. Products were sequenced in both forward and reverse directions on the Applied Biosystems 3500 DNA sequencer following the manufacturer’s instructions. All new sequences (n = 492) were deposited in GenBank (Accession Numbers: MT160874- MT161365, see Table S1 for details).

### Sequence alignment and phylogenetic reconstructions

All reliable sequences from additional populations and species of *Labeobarbus* from Ethiopia and Kenya (n = 138) were retrieved from GenBank for comprehensive analyses (Table S2). *Arabibarbus grypus* (KF876026), *L. beso* (AF180862), *L. nelspruitensis* (Gilchrist & Thompson 1911) (AF180866) as well as *L. ethiopicus* (AF180828) were selected as outgroups. All sequences were aligned and edited using Clustal X^94^ as implemented in MEGA v. 7.0^95^. The final dataset included 833 *cytb* sequences from all main drainages of Ethiopia, including Lake Tana.

The sequences were collapsed into common haplotypes using ALTER software^96^. DAMBE software^97^ was used to analyze substitution saturation by calculating the entropy-based index of substitution saturation (Iss) and its critical value (Iss.c)^98^. The whole dataset was tested first, and then again with only the third codon positions. In both cases, the Iss values were significantly lower than the Iss.c (p < 0.0001), indicating no or little substitution saturation, hence the data were suitable for phylogenetic inference.

Bayesian phylogenetic inference (BI) was performed using MrBayes v.3.2.6^99^. Two simultaneous runs with four Markov chains each were run for 1 × 10^7^ generations, sampled every 500 generations. The first 25% of runs were discarded as burn-in. Convergence of the runs was assessed by examining the average standard deviation of split frequencies and the potential scale reduction factor. In addition, stationarity was confirmed by examining posterior probability, log likelihood, and all model parameters by the effective sample sizes (ESSs) in the program Tracer v1.6^100^. The best‐fit model of molecular evolution by codon position for BI was estimated via the Bayesian information criterion using PartitionFinder v. 2.1.1^101–103^. The BI model used the following: 1st codon position K80 + I + G, 2nd codon position HKY, 3rd codon position GTR + G. The maximum likelihood (ML) search was performed using IQ-TREE 1.6.12^104^. The best partition scheme for ML was selected in ModelTest^105^ as implemented in IQ-TREE. Node support values were inferred using 1 000 bootstrap replicates. The ML model used the following: 1st codon position TIM3e + R2, 2nd codon position HKY + F, 3rd codon position GTR + F + G4.

The phylogenetic trees from the ML and BI analyses were visualized and edited using FigTree v.1.4.4^106^. In addition, a haplotype network was built with PopART 1.7 software^107^ using the median joining algorithm^108^.

Bayes factor (BF) comparisons of constrained and unconstrained tree topologies were used to test for monophyly of the riverine radiations. MrBayes was used to calculate the harmonic mean estimator of marginal likelihood for trees with a hard constraint of monophyly of the examined group. Bayes factors were calculated as the difference of harmonic mean estimators of the two models (constrained vs. unconstrained) in log units. According to Kass & Raftery^109^ a log difference of 3–5 is strong evidence, and a difference of > 5 is very strong evidence in favor of the better model. BI of constrained topologies were run with the same settings as described above for unconstrained phylogenetic analysis.

### Genetic diversity and structure

We calculated the number of haplotypes (*H*), haplotype diversity (*h*), nucleotide diversity (*π*), and the average number of nucleotide substitutions (*K*) for each supposed adaptive radiation and geographical population (by basin) using DnaSP v.5.10^110^. Genetic differentiation among sampling locations was tested in Arlequin v. 3.5.2.2^111^ using the fixation index F_ST_^112^ based on pairwise differences or haplotype frequencies. In addition, an exact test of sample differentiation (global and pairwise), which tests the non-random distribution of haplotypes into population samples under the hypothesis of panmixia, was calculated in Arlequin v. 3.5.2.2.

## Supplementary information

Supplementary Information.

## References

[1] Schluter, D. *The ecology of adaptive radiation* (OUP Oxford, 2000).

[2] Sturmbauer C (1998). Explosive speciation in cichlid fishes of the African Great Lakes: a dynamic model of adaptive radiation. J. Fish Biol..

[3] Kocher TD (2004). Adaptive evolution and explosive speciation: the cichlid fish model. Nat. Rev. Genet..

[4] Barluenga M, Stölting KN, Salzburger W, Muschick M, Meyer A (2006). Sympatric speciation in Nicaraguan crater lake cichlid fish. Nature.

[5] Losos JB, Ricklefs RE (2009). Adaptation and diversification on islands. Nature.

[6] Stroud JT, Losos JB (2016). Ecological opportunity and adaptive radiation. Annu. Rev. Ecol. Evol. Syst..

[7] Johns GC, Avise JC (1998). A comparative summary of genetic distances in the vertebrates from the mitochondrial cytochrome *b* gene. Mol. Biol. Evol..

[8] Seehausen O (2000). Explosive speciation rates and unusual species richness in haplochromine cichlid fishes: effects of sexual selection. Adv. Ecol. Res.

[9] Rüber L, Zardoya R (2005). Rapid cladogenesis in marine fishes revisited. Evolution.

[10] Puebla O (2009). Ecological speciation in marine v. freshwater fishes. J. Fish Biol.

[11] Matschiner M, Hanel R, Salzburger W (2011). On the origin and trigger of the notothenioid adaptive radiation. PLoS ONE.

[12] Seehausen O, Wagner CE (2014). Speciation in freshwater fishes. Annu. Rev. Ecol. Evol. Syst..

[13] Skúlason, S. Sympatric morphs, populations and speciation in freshwater fish with emphasis on arctic charr in *Evolution of Biological Diversity* (ed. Magurran, A & May, R. M.) 71-92 (Oxford University Press, 1999).

[14] Salzburger W, Meyer A (2004). The species flocks of East African cichlid fishes: recent advances in molecular phylogenetics and population genetics. Naturwissenschaften.

[15] Golubtsov, A. S. Fish ‘Species Flocks’ in Rivers and Lakes: Sympatric Divergence in Poor Fauna Fish Communities as Particular Modus of Evolution in *Relevant Problems of Contemporary Ichthyology* (ed. Pavlov, D. S., Dgebuadze, Y. Y., & Shatunovsky, M. I.) 96-123 (KMK, 2010).

[16] Sullivan JP, Lavoué S, Hopkins CD (2002). Discovery and phylogenetic analysis of a riverine species flock of African electric fishes (Mormyridae: Teleostei). Evolution.

[17] Feulner PGD, Kirschbaum F, Mamonekene V, Ketmaier V, Tiedemann R (2007). Adaptive radiation in African weakly electric fish (Teleostei: Mormyridae: *Campylomormyrus*): a combined molecular and morphological approach. J. Evol. Biol.

[18] Feulner PG, Kirschbaum F, Tiedemann R (2008). Adaptive radiation in the Congo River: an ecological speciation scenario for African weakly electric fish (Teleostei; Mormyridae; *Campylomormyrus*). J. Physiol.-Paris.

[19] Day JJ (2013). Continental diversification of an African catfish radiation (Mochokidae: *Synodontis*). Syst. Biol.

[20] Levin BA (2019). Adaptive radiation of barbs of the genus *Labeobarbus* (Cyprinidae) in an East African river. Freshwater Biol..

[21] Joyce DA (2005). An extant cichlid fish radiation emerged in an extinct Pleistocene lake. Nature.

[22] Koblmüller S (2008). Age and spread of the haplochromine cichlid fishes in Africa. Mol. Phylogenet. Evol..

[23] Kullander SO, Norén M, Friðriksson GB (2010). & Santos de Lucena, C. A. Phylogenetic relationships of species of *Crenicichla* (Teleostei: Cichlidae) from southern South America based on the mitochondrial cytochrome *b* gene. J. Zool. Syst. Evol. Res..

[24] Schwarzer J, Misof B, Ifuta SN, Schliewen UK (2011). Time and origin of cichlid colonization of the lower Congo rapids. PloS ONE.

[25] Piálek L, Říčan O, Casciotta J, Almirón A, Zrzavý J (2012). Multilocus phylogeny of *Crenicichla* (Teleostei: Cichlidae), with biogeography of the *C. lacustris* group: species flocks as a model for sympatric speciation in rivers. Mol. Phylogenet. Evol..

[26] Piálek L (2019). Phylogenomics of pike cichlids (Cichlidae: *Crenicichla*) of the *C. mandelburgeri* species complex: rapid ecological speciation in the Iguazú River and high endemism in the Middle Paraná basin. Hydrobiologia.

[27] Burress ED (2018). Island-and lake-like parallel adaptive radiations replicated in rivers. Proc. R. Soc. Lond. B Biol. Sci..

[28] Berg, L. S. *Fishes (Marsipobranchii and Pisces). Vol 3. Ostariophysi* (Imperial Academy of Sciences, 1914).

[29] Burnashev MS (1952). Snow trouts of the Zeravshan River. Proc. Kishinev State Univ. (Biol.).

[30] Roberts TR (1998). Review of the tropical Asian cyprinid fish genus *Poropuntius*, with descriptions of new species and trophic morphs. Nat. Hist. Bull. Siam Soc..

[31] Roberts TR, Khaironizam MZ (2008). Trophic polymorphism in the Malaysian fish *Neolissochilus soroides* and other old world barbs (Teleostei, Cyprinidae). Nat. Hist. Bull. Siam Soc..

[32] Golubtsov AS, Cherenkov SE, Tefera F (2012). High morphological diversity of the genus *Garra* in the Sore River (the White Nile Basin, Ethiopia): one more cyprinid species flock?. J. Ichthyol..

[33] Kullander SO, Roberts TR (2011). Out of Lake Tanganyika: endemic lake fishes inhabit rapids of the Lukuga River. Ichthyol. Explor. Freshwaters.

[34] Gillespie RG, Benjamin SP, Brewer MS, Rivera MAJ, Roderick GK (2018). Repeated diversification of ecomorphs in Hawaiian stick spiders. Curr. Biol..

[35] Colosimo PF (2005). Widespread parallel evolution in sticklebacks by repeated fixation of ectodysplasin alleles. Science.

[36] Losos JB (2011). Convergence, adaptation, and constraint. Evolution.

[37] Rüber L, Verheyen E, Meyer A (1999). Replicated evolution of trophic specializations in an endemic cichlid fish lineage from Lake Tanganyika. Proc. Nat. Acad. Sci..

[38] Elmer KR (2014). Parallel evolution of Nicaraguan crater lake cichlid fishes via non-parallel routes. Nat. Commun..

[39] Thompson CE, Taylor EB, McPhail JD (1997). Parallel evolution of lake‐stream pairs of threespine sticklebacks (*Gasterosteus*) inferred from mitochondrial DNA variation. Evolution.

[40] DeFaveri J, Shikano T, Shimada Y, Goto A, Merilä J (2011). Global analysis of genes involved in freshwater adaptation in threespine sticklebacks (*Gasterosteus aculeatus*). Evolution.

[41] Xie KT (2019). DNA fragility in the parallel evolution of pelvic reduction in stickleback fish. Science.

[42] Alekseyev SS, Samusenok VP, Matveev AN, Pichugin MY (2002). Diversification, sympatric speciation, and trophic polymorphism of Arctic charr, *Salvelinus alpinus* complex, in Transbaikalia. Env. Biol. Fish.

[43] Knudsen R, Klemetsen A, Alekseyev S, Adams CE, Power M (2016). The role of *Salvelinus* in contemporary studies of evolution, trophic ecology and anthropogenic change. Hydrobiologia.

[44] Reshetnikov, Y. S. *Ecology and systematics of whitefishes* (Nauka, 1980).

[45] Derome N, Bernatchez L (2006). The transcriptomics of ecological convergence between 2 limnetic coregonine fishes (Salmonidae). Mol. Biol. Evol..

[46] Østbye K (2006). Parallel evolution of ecomorphological traits in the European whitefish *Coregonus lavaretus* (L.) species complex during postglacial times. Mol. Ecol.

[47] Oellermann LK, Skelton PH (1990). Hexaploidy in yellowfish species (*Barbus*, Pisces, Cyprinidae) from southern Africa. J. Fish Biol..

[48] Golubtsov AS, Krysanov EY (1993). Karyological study of some cyprinid species from Ethiopia. The ploidy differences between large and small *Barbus* of Africa. J. Fish Biol..

[49] Naran D, Skelton PH, Villet MH (2007). Karyology of three evolutionarily hexaploid southern African species of yellowfish, *Labeobarbus* Rüppel, 1836 (Cyprinidae). Afr. Zool..

[50] Yang L (2015). Phylogeny and polyploidy: resolving the classification of cyprinine fishes (Teleostei: Cypriniformes). Mol. Phylogenet. Evol..

[51] Snoeks, J., Harrison, I. J., Stiassny, M. L. J. The status and distribution of freshwater fishes in *The diversity of life in African freshwaters: under water, under threat. An analysis of the status and distribution of freshwater species throughout mainland Africa* (ed. Darwall, W. R. T. *et al*.) 42-73 (IUCN, 2011).

[52] Vreven EJ, Musschoot T, Snoeks J, Schliewen UK (2016). The African hexaploid Torini (Cypriniformes: Cyprinidae): review of a tumultuous history. *Zool*. J. Linn. Soc..

[53] Beshera KA, Harris PM, Mayden RL (2016). Novel evolutionary lineages in *Labeobarbus* (Cypriniformes; Cyprinidae) based on phylogenetic analyses of mtDNA sequences. Zootaxa.

[54] Stewart KM, Murray AM (2017). Biogeographic implications of fossil fishes from the Awash River, Ethiopia. J. Vert. Paleontol.

[55] Groenewald AAVJ (1958). A revision of the genera *Barbus* and *Varicorhinus* in Transvaal. Ann. Transvaal Mus.

[56] Jubb, R. A. *Freshwater Fishes of Southern Africa* (Balkema, 1967).

[57] Banister KE (1973). A revision of the large *Barbus* (Pisces, Cyprinidae) of East and Central Africa: II. Studies of African Cyprinidae. Bull Brit. Mus. Nat. Hist. (Zool.).

[58] Banister KE (1984). Three new species of *Varicorhinus* (Pisces, Cyprinidae) from. Africa. Bull Brit. Mus. Nat. Hist. (Zool.).

[59] Nagelkerke LA, Sibbing FA, van den Boogaart JG, Lammens EH, Osse JW (1994). The barbs (Barbus spp.) of Lake Tana: a forgotten species flock? Env. Biol. Fish..

[60] Nagelkerke LAJ (2015). Shallow genetic divergence and species delineations in the endemic *Labeobarbus* species flock of Lake Tana, Ethiopia. J. Fish Biol..

[61] Mina MV, Mironovsky AN, Golubtsov AS, Dgebuadze YY (1998). The ‘*Barbus*’ *intermedius* species flock in Lake Tana (Ethiopia): II-Morphological diversity of” large barbs” from Lake Tana and neighbouring areas: Homoplasies or synapomorphies?. Ital. J. Zool..

[62] Mina MV, Mironovsky AN, Golani D (2001). Consequences and modes of morphological diversification of East African and Eurasian barbins (genera *Barbus*, *Varicorhinus* and *Capoeta*) with particular reference to *Barbus intermedius* complex. Env. Biol. Fish.

[63] Dimmick WW, Berendzen PB, Golubtsov AS (2001). Genetic comparison of three *Barbus* (Cyprinidae) morphotypes from the Genale River, Ethiopia. Copeia.

[64] de Graaf M, Dejen E, Osse JW, Sibbing FA (2008). Adaptive radiation of lake Tana’s (Ethiopia) *Labeobarbus* species flock (Pisces, Cyprinidae). Mar. Freshwater Res..

[65] de Graaf M, Megens HJ, Samallo J, Sibbing F (2010). Preliminary insight into the age and origin of the *Labeobarbus* fish species flock from Lake Tana (Ethiopia) using the mtDNA cytochrome *b* gene. Mol. Phylogenet. Evol..

[66] Tsigenopoulos CS, Kasapidis P, Berrebi P (2010). Phylogenetic relationships of hexaploid large-sized barbs (genus *Labeobarbus*, Cyprinidae) based on mtDNA data. Mol. Phylogenet. Evol..

[67] Levin BA (2012). New data on morphology of the African scraping feeder *Varicorhinus beso* (Osteichthyes: Cyprinidae) with the special reference to specialized traits. J. Ichthyol.

[68] Levin BA, Golubtsov AS, Dgebuadze YY, Mugue NS (2013). New evidence of homoplasy within the African genus *Varicorhinus* (Cyprinidae): an independent origin of specialized scraping forms in the adjacent drainage systems of Ethiopia inferred from mtDNA analysis. Afr. Zool..

[69] Vreven EJ (2019). The complex origins of mouth polymorphism in the *Labeobarbus* (Cypriniformes: Cyprinidae) of the Inkisi River basin (Lower Congo, DRC, Africa): insights from an integrative approach. *Zool*. J. Linn. Soc..

[70] Mironovsky AN, Mina MV, Dgebuadze YY (2019). Large African Barbs with Hypertrophied Lips and their Relationship with Generalized Forms of Species of the Genus *Barbus* (*Labeobarbus* auctorum). J. Ichthyol..

[71] Nagelkerke LA, Mina MV, Wudneh T, Sibbing FA, Osse JW (1995). In Lake Tana, a Unique Fish Fauna Needs Protection: These Ethiopian barbs constitute the only cyprinid species flock known that has not been damaged by human influences. Bioscience.

[72] Beshera KA, Harris PM, Mitochondrial DNA (2014). phylogeography of the *Labeobarbus intermedius* complex (Pisces, Cyprinidae) from Ethiopia. J. Fish Biol..

[73] Herre AW (1933). The fishes of Lake Lanao: A problem in evolution. Amer. Nat..

[74] Kornfield, I., & Carpenter, K. E. Cyprinids of Lake Lanao, Philippines: taxonomic validity, evolutionary rates and speciation scenarios in *Еvolution of Species Flocks* (ed. Eshelle, A. A. & Kornfield, I.) 69-83 (Orono Press, 1984).

[75] Golubtsov AS (1993). Biogéographie des ‘grands *Barbus*’ d’Éthiopie avec référence spéciale à des formes à statuts taxinomiques incertains. Cah. Ethol.

[76] Hudson AG, Vonlanthen P, Seehausen O (2011). Rapid parallel adaptive radiations from a single hybridogenic ancestral population. Proc. Royal Soc. B Biol. Sci..

[77] Markevich G, Esin E, Anisimova L (2018). Basic description and some notes on the evolution of seven sympatric morphs of Dolly Varden *Salvelinus malma* from the Lake Kronotskoe Basin. Ecol. Evol.

[78] Schön I, Martens K (2004). Adaptive, pre-adaptive and non-adaptive components of radiations in ancient lakes: a review. Organ. Divers. Evol.

[79] Ferguson DJ (2010). Recent rift-related volcanism in Afar, Ethiopia. Earth Planet. Sci. Let.

[80] Hutchison W (2016). The eruptive history and magmatic evolution of Aluto volcano: new insights into silicic peralkaline volcanism in the Ethiopian rift. J. Volcanol. Geotherm. Res..

[81] Prave AR (2016). Geology and geochronology of the Tana Basin, Ethiopia: LIP volcanism, super eruptions and Eocene–Oligocene environmental change. Earth Planet. Sci. Let.

[82] Ayvazyan A, Vasilyan D, Böhme M (2019). Possible species-flock scenario for the evolution of the cyprinid genus *Capoeta* (Cypriniformes: Cyprinidae) within late Neogene lake systems of the Armenian Highland. PloS ONE.

[83] Selz OM, Seehausen O (2019). Interspecific hybridization can generate functional novelty in cichlid fish. Proc. Royal Soc. B Biol. Sci..

[84] Marques DA, Meier JI, Seehausen O (2019). A combinatorial view on speciation and adaptive radiation. Trends Ecol. Evol..

[85] Genner MJ, Turner GF (2012). Ancient hybridization and phenotypic novelty within Lake Malawi’s cichlid fish radiation. Mol. Biol. Evol..

[86] Meier JI (2017). Ancient hybridization fuels rapid cichlid fish adaptive radiations. Nature Commun..

[87] Irisarri I (2018). Phylogenomics uncovers early hybridization and adaptive loci shaping the radiation of Lake Tanganyika cichlid fishes. Nature Commun..

[88] Borkenhagen K (2014). A new genus and species of cyprinid fish (Actinopterygii, Cyprinidae) from the Arabian Peninsula, and its phylogenetic and zoogeographic affinities. Env. Biol. Fish.

[89] Coad, B. Freshwater Fishes of Iran. Retrieved from http://www.briancoad.com/Species%20Accounts/Contents%20new.htm (2019).

[90] Losos, J. B., & Mahler, D. L. Adaptive radiation: the interaction of ecological opportunity, adaptation, and speciation in *Evolution since Darwin: the first 150 years* (ed. Bell, M. A.) 381-420 (Sinauer Associates, 2010).

[91] Aljanabi SM, Martinez I (1997). Universal and rapid salt-extraction of high quality genomic DNA for PCR-based techniques. Nucl. Acids Res.

[92] Palumbi, S. R. Nucleic acids II: The polymerase chain reaction in *Molecular systematics* (ed. Hillis, D. M., Moritz, C., & Mable, B. K.) 205-247 (Sinauer Associates, 1996).

[93] Perdices A, Doadrio I (2001). The molecular systematics and biogeography of the European cobitids based on mitochondrial DNA sequences. Mol. Phylogenet. Evol..

[94] Thompson JD, Higgins DG, Gibson TJ (1994). CLUSTAL W: improving the sensitivity of progressive multiple sequence alignment through sequence weighting, position-specific gap penalties and weight matrix choice. Nucl. Acids Res.

[95] Kumar S, Stecher G, Tamura K (2016). MEGA7: molecular evolutionary genetics analysis version 7.0 for bigger datasets. Mol. Biol. Evol..

[96] Glez‐Peña D, Gómez‐Blanco D, Reboiro‐Jato M, Fdez‐Riverola F, Posada D (2010). ALTER: Program‐oriented format conversion of DNA and protein alignments. Nucl. Acids Res.

[97] Xia X (2018). DAMBE7: new and improved tools for data analysis in molecular biology and evolution. Mol. Biol. Evol..

[98] Xia X, Xie Z, Salemi M, Chen L, Wang Y (2003). An index of substitution saturation and its application. Mol. Phylogenet. Evol..

[99] Ronquist F (2012). MrBayes 3.2: Efficient Bayesian phylogenetic inference and model choice across a large model space. Syst. Biol.

[100] Rambaut, A., Suchard, M. A., Xie, D. & Drummond, A. J. Tracer v1.6. Retrieved from http://beast.bio.ed.ac.uk/Tracer (2014).

[101] Lanfear R, Calcott B, Ho SY, Guindon S (2012). PartitionFinder: combined selection of partitioning schemes and substitution models for phylogenetic analyses. Mol. Biol. Evol..

[102] Lanfear R, Frandsen PB, Wright AM, Senfeld T, Calcott B (2017). PartitionFinder 2: new methods for selecting partitioned models of evolution for molecular and morphological phylogenetic analyses. Mol. Biol. Evol..

[103] Guindon S (2010). New algorithms and methods to estimate maximum-likelihood phylogenies: assessing the performance of PhyML 3.0. Syst. Biol.

[104] Nguyen LT, Schmidt HA, Von Haeseler A, Minh BQ (2015). IQ-TREE: a fast and effective stochastic algorithm for estimating maximum-likelihood phylogenies. Mol. Biol. Evol.

[105] Kalyaanamoorthy S, Minh BQ, Wong TK, von Haeseler A, Jermiin LS (2017). ModelFinder: fast model selection for accurate phylogenetic estimates. Nature Methods.

[106] Rambaut, A. FigTree 1.4.2 software. *Institute of Evolutionary Biology, Univ. Edinburgh*. (2014).

[107] Leigh JW, Bryant D (2015). Popart: full‐feature software for haplotype network construction. Methods Ecol. Evol.

[108] Bandelt HJ, Forster P, Röhl A (1999). Median-joining networks for inferring intraspecific phylogenies. Mol. Biol. Evol..

[109] Kass RE, Raftery AE (1995). Bayes factors. J. Amer. Stat. Associat.

[110] Librado P, Rozas J (2009). DnaSP v5: a software for comprehensive analysis of DNA polymorphism data. Bioinformatics.

[111] Excoffier L, Lischer HE (2010). Arlequin suite ver 3.5: a new series of programs to perform population genetics analyses under Linux and Windows. Mol. Ecol. Res.

[112] Weir BS, Cockerham CC (1984). Estimating F‐statistics for the analysis of population structure. Evolution.

