# Supplementary material for: In the rivers: Multiple adaptive radiations of cyprinid fishes (Labeobarbus) in Ethiopian Highlands
Source: Sci Rep. 2020 Apr 28;10:7192. doi: 10.1038/s41598-020-64350-4 (PMC7189375; doi:10.1038/s41598-020-64350-4)
Supplement: Supplementary file 1 — Supplementary Information. [file 41598_2020_64350_MOESM1_ESM.docx]

Supplementary information

**In the rivers: Multiple adaptive radiations of cyprinid fishes (*Labeobarbus*) in Ethiopian Highlands**

Boris A. Levin^1,2*^, Evgeniy Simonov^3,4^, Yury Y. Dgebuadze^5^, Marina Levina^1^, Alexander S. Golubtsov^5^

^1^ Papanin Institute of Biology of Inland Waters, Russian Academy of Sciences, Borok, Russia

^2^ Cherepovets State University, Cherepovets, Russia

^3^ Institute of Environmental and Agricultural Biology (X-BIO),
University of Tyumen, Tyumen, Russia

^4^ Tomsk State University, Russia

^5^ Severtsov Institute of Ecology and Evolution, Russian Academy of Sciences, Moscow, Russia

* Corresponding author–borislyovin@gmail.com


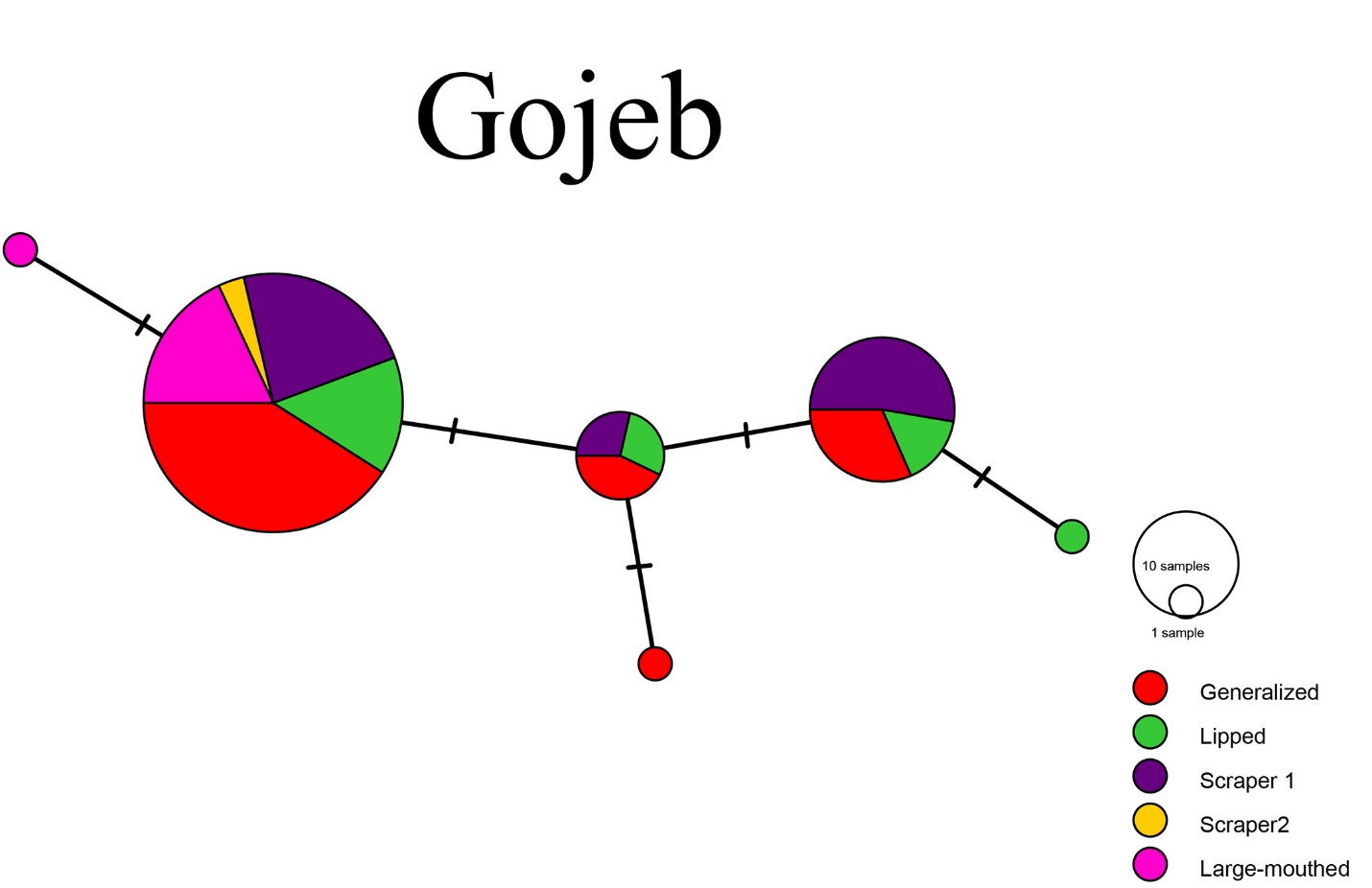


Fig. S1. Median-joining haplotype network of *Labeobarbus* from the Gojeb River (Omo-Turkana basin), constructed based on *cytb* sequences.


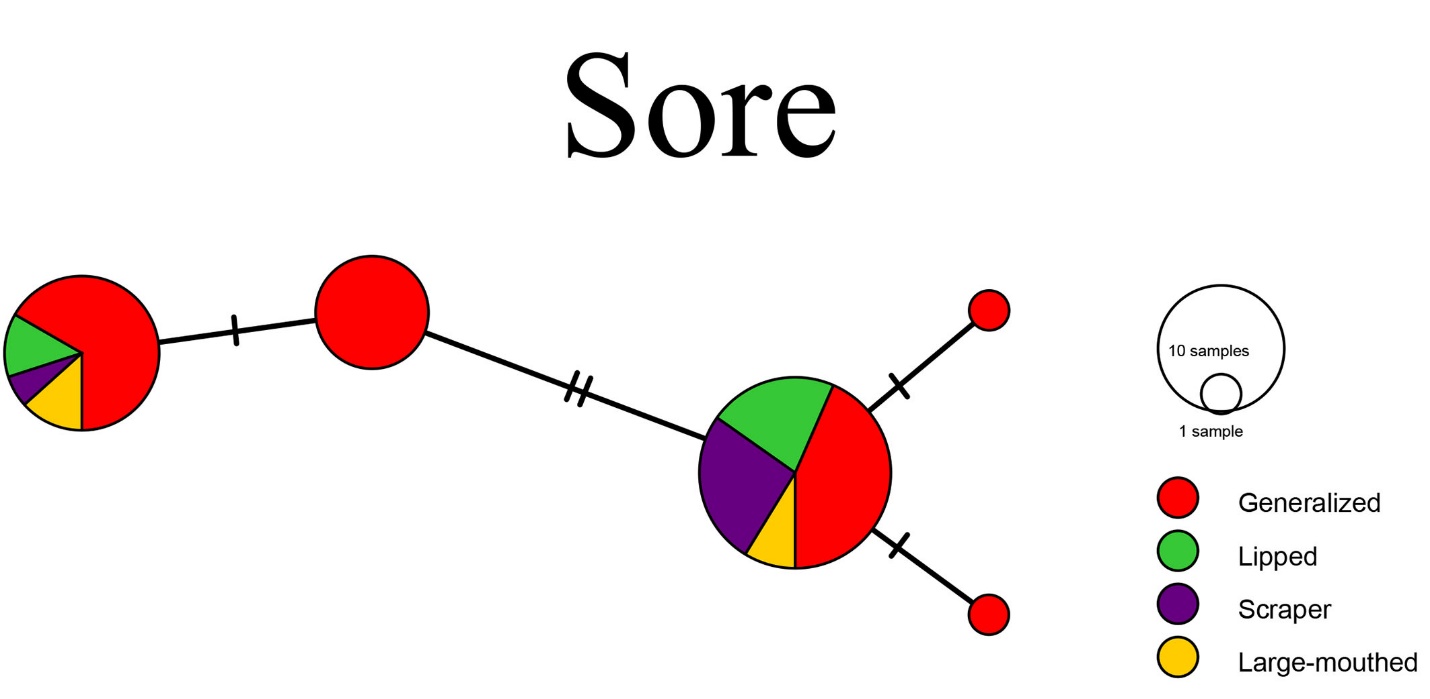


Fig. S2. Median-joining haplotype network of *Labeobarbus* from the Sore River (White Nile basin), constructed based on *cytb* sequences.


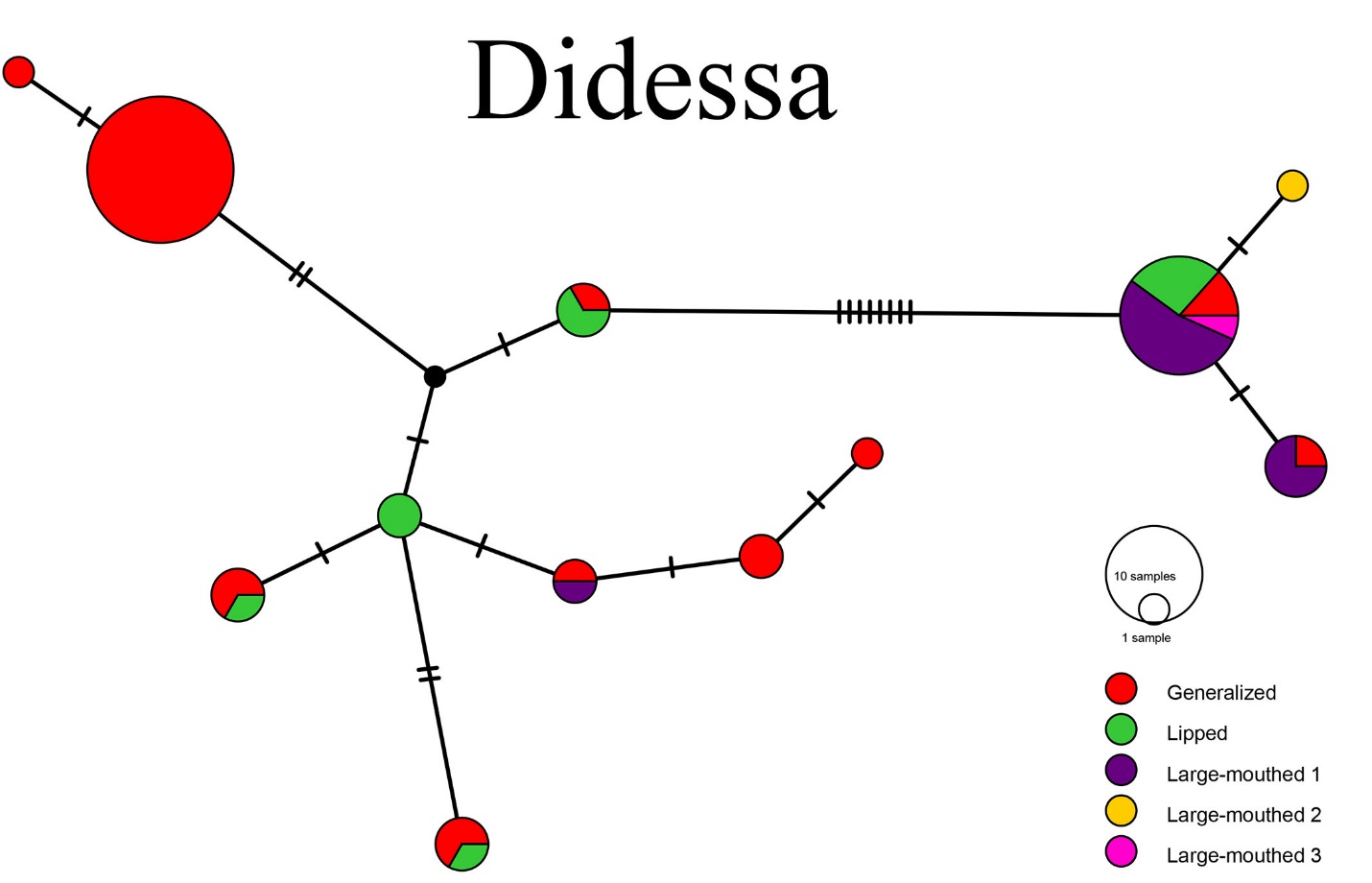


Fig. S3. Median-joining haplotype network of *Labeobarbus* from the Didessa River (Blue Nile nasin), constructed based on *cytb* sequences. Black dot represents hypothetical intermediate haplotypes.

Table S1. Localities and DNA samples of *Labeobarbus* spp. from Ethiopian water bodies used in this study.

| No. | Locality | Basin | Geographical coordinates | DNA sample size | GenBank Nos. |
| --- | --- | --- | --- | --- | --- |
| **Indian Ocean catchment** | | | | | |
| 1* | Genale River | Juba basin, | 05.7023N 39.5442E | 153 | MK001027-MK001180 |
| 2 | Welmel River | Genale R., Juba basin | 06.2261N 39.8195E | 7 | JQ701761-62; MK001388-92 |
| 3 | Dawa River | Juba basin | 05.3283N 38.7869E | 11 | MK001185-95 |
| 4 | Awata River | Dawa R., Juba basin | 05.7852N 38.9294E | 9 | MK001196- MK001204 |
| 5 | Weyb River | Wabe-Shebelle basin | 06.8983N 40.8516E | 2 | MK001182-83 |
| 6 | Burka River | Wabe-Shebelle basin | 07.4963N 40.9813E | 10 | MK001184, MT160972- MT160980 |
| 7 | Wabe River | Wabe-Shebelle basin | 07.4547N 40.8516E | 4 | MK015650-52, MT161365 |
| 8 | Shebelle River | Wabe-Shebelle basin | 07.9884N 40.4424E | 2 | MT161301-02 |
| **Ethiopian Rift Valley** | | | | | |
| 9 | Awash River at Sodore | Awash basin | 08.3911N 39.3967E | 6 | MT160905-10 |
| 10 | Koka Reservoir | Awash basin | 8.4053N 39.0209E | 1 | MT160904 |
| 11 | Awash River at Adaitu | Awash basin | 11.1271N 40.7609E | 4 | MT160900-03 |
| 12 | Lake Awassa | Lake Awassa | 7.0421N 38.4570E | 20 | MT160911- MT160930 |
| 13 | Lake Langano | Lake Langano | 7.5513N 38.6835E | 9 | MT161249- MT161257 |
| 14 | Lepis River | Lake Langano | 07.5166N 37.7500E | 2 | MT161268-69 |
| 15 | Huluka River | Lake Langano | 07.5000N 37.7333E | 10 | MT161258- MT161267 |
| 16 | Raya River | Lake Abaya | 06.4608N 37.7411E | 16 | MT161270- MT161285 |
| 17 | Hare River | Lake Abaya | 06.0581N 37.6001E | 5 | MT161244-48 |
| 18 | Sile River | Lake Chamo | 05.9009N 37.5033E | 12 | MT161303- MT161314 |
| 19 | Sago River | Lake Chamo | 05.8930N 37.4277E | 10 | MT161286- MT161295 |
| 20 | Sagan River | Lake Chew Bahir | 05.2347N 37.5244E | 5 | MT161296- MT161300 |
| **Lake Tana and Blue Nile drainage** | | | | | |
| 21* | Didessa River | Blue Nile | 08.6835N 36.4166E | 10 | MT160997- MT161006 |
| 22* | Didessa River | Blue Nile | 08.0446N 36.4738E | 50 | MT161007- MT161056 |
| 23 | Dabena River | Didessa River | 08.4062N 36.2900E | 5 | MT160981-85 |
| 24 | Enfras River | Lake Tana | 11.6222N 37.2896E | 7 | MT161057- MT161063 |
| **White Nile** | | | | | |
| 25* | Sore River | Baro drainage | 08.3214N 35.5962E | 26 | MT161315- MT161340 |
| 26* | Sore River | Baro drainage | 08.3973N 35.4352E | 23 | MT161341- MT161364 |
| 27 | Birbir River upstream | Baro drainage | 08.7358N 35.3529E | 28 | MT160943- MT160970 |
| 28 | Birbir River close to mouth | Baro drainage | 8.2484N 34.9654E | 5 | MT160938- MT160942 |
| 29 | Gabba River at bridge Metu-Supe | Baro drainage | 08.4821N 35.6462E | 20 | MT161074- MT161093 |
| 30 | Gabba River upstream | Baro drainage | 08.3614N 36.0411E | 10 | MT161064- MT161073 |
| 31 | Geba River | Baro drainage | 08.2198N 34.9679E | 6 | MT161094-99 |
| 32 | Baro River | Sobat drainage | 08.2454N 34.5887E | 4 | MT160931-34 |
| **Nile** | | | | | |
| 33 | Angereb River | Atbara basin | 13.1906N 37.2256E | 11 | MT160889-MT160899 |
| 34 | Angereb River | Atbara basin | 13.5167N 36.5000E | 11 | MT160878-MT160888 |
| 35 | Bebew River | Angereb basin | 13.1587N 37.2400E | 3 | MT160935-37 |
| 36 | Agula River | Genfel basin | 13.6886N 39.5793E | 4 | MT160874-77 |
| **Omo-Turkana drainage** | | | | | |
| 37* | Gojeb River at Chida | Gibe basin | 07.2541N 36.7944E | 123 | MT161118-MT161241 |
| 38 | Gibe River at Bako | Omo basin | 09.1266N 37.0480E | 5 | MT161104-08 |
| 39 | Gibe River at Welkite | Omo basin | 08.2439N 37.5495E | 4 | MT161100-03 |
| 40 | Gilgel-Gibe | Gibe basin | 07.7599N 37.1936N | 9 | MT161109-MT161117 |
| 41 | Lake Turkana | Lake Turkana | 04.4549N 36.2122E | 2 | MT161242-43 |

* - localities, where presumed riverine radiations detected.

Table S2. Comparative material retrieved from GenBank (<https://www.ncbi.nlm.nih.gov>)

| GenBank Accession nos. | Locality | Source |
| --- | --- | --- |
| *Labeobarbus altianalis* | | |
| MH484560-61 | Awach River, Lake Victoria basin, Kenya | Ndeda *et al.*, 2018^1^ |
| MH484562 | Nzoia Nyadorera River, Lake Victoria basin, Kenya | Ndeda *et al.*, 2018^1^ |
| MH484563 | Oluchi River, Lake Victoria basin, Kenya | Ndeda *et al.*, 2018^1^ |
| MH484602 | Nzoia River, Lake Victoria basin, Kenya | Ndeda *et al.*, 2018^1^ |
| MH484603 | Awach seme River, Lake Victoria basin, Kenya | Ndeda  *et al.*, 2018^1^ |
| *Labeobarbus intermedius* | | |
| GQ853236 | Lake Tana, Ethiopia | de Graaf  *et al.*, 2010^2^ |
| GQ853237 | Lake Tana, Ethiopia | de Graaf  *et al.*, 2010^2^ |
| GQ853238 | Lake Tana, Ethiopia | de Graaf  *et al.*, 2010^2^ |
| GQ853239 | Lake Chamo, Ethiopia | de Graaf  *et al.*, 2010^2^ |
| GQ853240 | Lake Chamo, Ethiopia | de Graaf  *et al.*, 2010^2^ |
| GQ853241 | Lake Chamo, Ethiopia | de Graaf  *et al.*, 2010^2^ |
| GQ853242 | Lake Chamo, Ethiopia | de Graaf  *et al.*, 2010^2^ |
| GQ853243 | Lake Awassa, Ethiopia | de Graaf  *et al.*, 2010^2^ |
| GQ853244 | Lake Awassa, Ethiopia | de Graaf  *et al.*, 2010^2^ |
| GQ853248 | Didessa River, Blue Nile basin, Ethiopia | de Graaf  *et al.*, 2010^2^ |
| GQ853249 | Didessa River, Blue Nile basin, Ethiopia | de Graaf  *et al.*, 2010^2^ |
| GQ853254 | Didessa River, Blue Nile basin, Ethiopia | de Graaf  *et al.*, 2010^2^ |
| GQ853255 | Didessa River, Blue Nile basin, Ethiopia | de Graaf  *et al.*, 2010^2^ |
| GQ853256 | Didessa River, Blue Nile basin, Ethiopia | de Graaf  *et al.*, 2010^2^ |
| GQ853257 | Didessa River, Blue Nile basin, Ethiopia | de Graaf  *et al.*, 2010^2^ |
| GQ853258 | Didessa River, Blue Nile basin, Ethiopia | de Graaf  *et al.*, 2010^2^ |
| GQ853259 | Didessa River, Blue Nile basin, Ethiopia | de Graaf  *et al.*, 2010^2^ |
| GQ853260 | Didessa River, Blue Nile basin, Ethiopia | de Graaf  *et al.*, 2010^2^ |
| GQ853261 | Didessa River, Blue Nile basin, Ethiopia | de Graaf  *et al.*, 2010^2^ |
| GQ853262 | Didessa River, Blue Nile basin, Ethiopia | de Graaf  *et al.*, 2010^2^ |
| GQ853263 | Didessa River, Blue Nile basin, Ethiopia | de Graaf  *et al.*, 2010^2^ |
| GQ853264 | Didessa River, Blue Nile basin, Ethiopia | de Graaf  *et al.*, 2010^2^ |
| GQ853265 | Didessa River, Blue Nile basin, Ethiopia | de Graaf  *et al.*, 2010^2^ |
| GQ853269 | Lugo River, tributary of Didessa R., Blue Nile basin, Ethiopia | de Graaf  *et al.*, 2010^2^ |
| JN886992 | Blue Nile, Ethiopia | Beshera & Harris, 2014^3^ |
| JN886993 | Blue Nile, Ethiopia | Beshera & Harris, 2014^3^ |
| JN886994 | Blue Nile, Ethiopia | Beshera & Harris, 2014^3^ |
| JN886995 | Blue Nile, Ethiopia | Beshera & Harris, 2014^3^ |
| JN886996 | Didessa River, Blue Nile basin, Ethiopia | Beshera & Harris, 2014^3^ |
| JN886997 | Didessa River, Blue Nile basin, Ethiopia | Beshera & Harris, 2014^3^ |
| JN886998 | Didessa River, Blue Nile basin, Ethiopia | Beshera & Harris, 2014^3^ |
| JN886999 | Didessa River, Blue Nile basin, Ethiopia | Beshera & Harris, 2014^3^ |
| JN887000 | Didessa River, Blue Nile basin, Ethiopia | Beshera & Harris, 2014^3^ |
| JN887001 | Didessa River, Blue Nile basin, Ethiopia | Beshera & Harris, 2014^3^ |
| JQ716387 | Didessa River, Blue Nile basin, Ethiopia | Beshera & Harris, 2014^3^ |
| JN887002 | Gumara River, Lake Tana basin, Ethiopia | Beshera & Harris, 2014^3^ |
| JN887004 | Gumara River, Lake Tana basin, Ethiopia | Beshera & Harris, 2014^3^ |
| JN887005 | Lake Tana, Ethiopia | Beshera & Harris, 2014^3^ |
| JN887006 | Lake Tana, Ethiopia | Beshera & Harris, 2014^3^ |
| JN887007 | Lake Tana, Ethiopia | Beshera & Harris, 2014^3^ |
| AF145948 | Lake Tana, Ethiopia | Durand *et al.*, 2002^4^ |
| AF287433 | Lake Tana, Ethiopia | Machordom & Doadrio, 2001^5^ |
| JN887008 | Awash River, Ethiopia | Beshera & Harris, 2014^3^ |
| JN887009 | Awash River, Ethiopia | Beshera & Harris, 2014^3^ |
| JN887010 | Lake Awassa, Ethiopia | Beshera & Harris, 2014^3^ |
| JN887011 | Lake Langano, Ethiopia | Beshera & Harris, 2014^3^ |
| JN887012 | Lake Langano, Ethiopia | Beshera & Harris, 2014^3^ |
| JN887013 | Lake Langano, Ethiopia | Beshera & Harris, 2014^3^ |
| JN887014 | Arba Minch spring, Abaya Lake basin, Ethiopia | Beshera & Harris, 2014^3^ |
| JN887015 | Arba Minch spring, Abaya Lake basin, Ethiopia | Beshera & Harris, 2014^3^ |
| JN887016 | Arba Minch spring, Abaya Lake basin, Ethiopia | Beshera & Harris, 2014^3^ |
| JN887017 | Arba Minch spring, Abaya Lake basin, Ethiopia | Beshera & Harris, 2014^3^ |
| JN887018 | Kulfo River, Chamo-Abaya lake system, Ethiopia | Beshera & Harris, 2014^3^ |
| JN887019 | Kulfo River, Chamo-Abaya lake system, Ethiopia | Beshera & Harris, 2014^3^ |
| JN887020 | Gibe River, Omo-Turkana basin, Ethiopia | Beshera & Harris, 2014^3^ |
| JN887021 | Gilgel Gibe, Omo-Turkana basin, Ethiopia | Beshera & Harris, 2014^3^ |
| JN887022 | Gilgel Gibe, Omo-Turkana basin, Ethiopia | Beshera & Harris, 2014^3^ |
| JN887023 | Gibe River, Omo-Turkana basin, Ethiopia | Beshera & Harris, 2014^3^ |
| JN887024 | Gojeb River, Omo-Turkana basin, Ethiopia | Beshera & Harris, 2014^3^ |
| JN887025 | Gojeb River, Omo-Turkana basin, Ethiopia | Beshera & Harris, 2014^3^ |
| JN887026 | Lake Chamo, Ethiopia | Beshera & Harris, 2014^3^ |
| JN887027 | Lake Chamo, Ethiopia | Beshera & Harris, 2014^3^ |
| JN887028 | Lake Abaya, Ethiopia | Beshera & Harris, 2014^3^ |
| KU524936 | Lumē Shet’ Awash basin, Ethiopia | Borkenhagen, 2017^6^ |
| KU524946 | Stream near Ejaji, Omo-Turkana basin, Ethiopia | Borkenhagen, 2017^6^ |
| KU524947 | Stream near Ejaji, Omo-Turkana basin, Ethiopia | Borkenhagen, 2017^6^ |
| KU524948 | Stream near Yayu, Gabba River tributary, White Nile basin, Ethiopia | Borkenhagen, 2017^6^ |
| KU524949 | Stream near Yayu, Gabba River tributary, White Nile basin, Ethiopia | Borkenhagen, 2017^6^ |
| KU524950 | Sore River, White Nile basin, Ethiopia | Borkenhagen, 2017^6^ |
| KU524951 | Sore River, White Nile basin, Ethiopia | Borkenhagen, 2017^6^ |
| KU524952 | Baro River, White Nile basin, Ethiopia | Borkenhagen, 2017^6^ |
| KU524953 | Baro River, White Nile basin, Ethiopia | Borkenhagen, 2017^6^ |
| KU524955 | Didessa River, Blue Nile basin, Ethiopia | Borkenhagen, 2017^6^ |
| AF112406 | n/a | Tsigenopoulos & Berrebi, 2000^7^ |
| AF180872 | Lake Baringo, Kenya | Tsigenopoulos *et al.*, 2010^8^ |
| AP011195 | n/a | Miya, 2009^9^ |
| NC031531 | n/a | Miya, 2009^9^ |
| *Labeobarbus acutirostris* | | |
| JN887030 | Lake Tana, Ethiopia | Beshera & Harris, 2014^3^ |
| GQ853203 | Lake Tana, Ethiopia | de Graaf  *et al.*, 2010^2^ |
| GQ853202 | Lake Tana, Ethiopia | de Graaf  *et al.*, 2010^2^ |
| GQ853201 | Lake Tana, Ethiopia | de Graaf  *et al.*, 2010^2^ |
| JQ716372 | Lake Tana, Ethiopia | Beshera & Harris, 2014^3^ |
| JQ716371 | Lake Tana, Ethiopia | Beshera & Harris, 2014^3^ |
| *Labeobarbus brevicephalus* | | |
| GQ853204 | Lake Tana, Ethiopia | de Graaf  *et al.*, 2010^2^ |
| GQ853205 | Lake Tana, Ethiopia | de Graaf  *et al.*, 2010^2^ |
| *Labeobarbus dainellii* | | |
| GQ853209 | Lake Tana, Ethiopia | de Graaf  *et al.*, 2010^2^ |
| GQ853210 | Lake Tana, Ethiopia | de Graaf  *et al.*, 2010^2^ |
| *Labeobarbus platydorsus* | | |
| JN887032 | Lake Tana, Ethiopia | Beshera & Harris, 2014^3^ |
| JN887034 | Lake Tana, Ethiopia | Beshera & Harris, 2014^3^ |
| JQ716374 | Lake Tana, Ethiopia | Beshera & Harris, 2014^3^ |
| JQ716375 | Lake Tana, Ethiopia | Beshera & Harris, 2014^3^ |
| JQ716382 | Lake Tana, Ethiopia | Beshera & Harris, 2014^3^ |
| GQ853211 | Lake Tana, Ethiopia | de Graaf  *et al.*, 2010^2^ |
| GQ853212 | Lake Tana, Ethiopia | de Graaf  *et al.*, 2010^2^ |
| GQ853213 | Lake Tana, Ethiopia | de Graaf  *et al.*, 2010^2^ |
| GQ853226 | Lake Tana, Ethiopia | de Graaf  *et al.*, 2010^2^ |
| GQ853227 | Lake Tana, Ethiopia | de Graaf  *et al.*, 2010^2^ |
| GQ853228 | Lake Tana, Ethiopia | de Graaf  *et al.*, 2010^2^ |
| *Labeobarbus gorgorensis* | | |
| JN887034 | Lake Tana, Ethiopia | Beshera & Harris, 2014^3^ |
| *Labeobarbus longissimus* | | |
| GQ853216 | Lake Tana, Ethiopia | de Graaf  *et al.*, 2010^2^ |
| *Labeobarbus macrocephalus* | | |
| GQ853217 | Lake Tana, Ethiopia | de Graaf  *et al.*, 2010^2^ |
| GQ853218 | Lake Tana, Ethiopia | de Graaf  *et al.*, 2010^2^ |
| GQ853219 | Lake Tana, Ethiopia | de Graaf  *et al.*, 2010^2^ |
| JQ716379 | Lake Tana, Ethiopia | Beshera & Harris, 2014^3^ |
| *Labeobarbus megastoma* | | |
| GQ853220 | Lake Tana, Ethiopia | de Graaf  *et al.*, 2010^2^ |
| GQ853221 | Lake Tana, Ethiopia | de Graaf  *et al.*, 2010^2^ |
| GQ853222 | Lake Tana, Ethiopia | de Graaf  *et al.*, 2010^2^ |
| JN887033 | Lake Tana, Ethiopia | Beshera & Harris, 2014^3^ |
| JQ716380 | Lake Tana, Ethiopia | Beshera & Harris, 2014^3^ |
| *Labeobarbus nedgia* | | |
| GQ853225 | Lake Tana, Ethiopia | de Graaf  *et al.*, 2010^2^ |
| GQ853224 | Lake Tana, Ethiopia | de Graaf  *et al.*, 2010^2^ |
| GQ853223 | Lake Tana, Ethiopia | de Graaf  *et al.*, 2010^2^ |
| JQ716378 | Lake Tana, Ethiopia | Beshera & Harris, 2014^3^ |
| JQ716377 | Lake Tana, Ethiopia | Beshera & Harris, 2014^3^ |
| JQ716376 | Lake Tana, Ethiopia | Beshera & Harris, 2014^3^ |
| JN887031 | Lake Tana, Ethiopia | Beshera & Harris, 2014^3^ |
| *Labeobarbus surkis* | | |
| GQ853229 | Lake Tana, Ethiopia | de Graaf  *et al.*, 2010^2^ |
| JQ716381 | Lake Tana, Ethiopia | Beshera & Harris, 2014^3^ |
| *Labeobarbus truttiformes* | | |
| GQ853230 | Lake Tana, Ethiopia | de Graaf  *et al.*, 2010^2^ |
| GQ853231 | Lake Tana, Ethiopia | de Graaf  *et al.*, 2010^2^ |
| GQ853232 | Lake Tana, Ethiopia | de Graaf  *et al.*, 2010^2^ |
| JQ716386 | Lake Tana, Ethiopia | Beshera & Harris, 2014^3^ |
| *Labeobarbus tsanensis* | | |
| GQ853233 | Lake Tana, Ethiopia | de Graaf  *et al.*, 2010^2^ |
| GQ853234 | Lake Tana, Ethiopia | de Graaf  *et al.*, 2010^2^ |
| GQ853235 | Lake Tana, Ethiopia | de Graaf  *et al.*, 2010^2^ |
| JQ716383 | Lake Tana, Ethiopia | Beshera & Harris, 2014^3^ |
| JQ716384 | Lake Tana, Ethiopia | Beshera & Harris, 2014^3^ |
| JQ716385 | Lake Tana, Ethiopia | Beshera & Harris, 2014^3^ |
| *Labeobarbus zaphiri* | | |
| AF180871 | Didessa River, Blue Nile basin, Ethiopia | Durand *et al.*, 2002^4^ |
| *Labeobarbus jubae* | | |
| JN887035 | Genale River, Juba ̶ Wabe-Shebelle basin | Beshera & Harris, 2014^3^ |
| *Labeobarbus gananensis* | | |
| JN887036 | Genale River, Juba ̶ Wabe-Shebelle basin | Beshera & Harris, 2014^3^ |
| JN887037 | Genale River, Juba ̶ Wabe-Shebelle basin | Beshera & Harris, 2014^3^ |
| JN887038 | Genale River, Juba ̶ Wabe-Shebelle basin | Beshera & Harris, 2014^3^ |

References

1. Ndeda, V. M., Mateos, M., & Hurtado, L. A. (2018). Evolution of African barbs from the Lake Victoria drainage system, Kenya. *PeerJ* **6**, e5762.

2. de Graaf, M., Megens, H. J., Samallo, J., & Sibbing, F. (2010). Preliminary insight into the age and origin of the *Labeobarbus* fish species flock from Lake Tana (Ethiopia) using the mtDNA cytochrome *b* gene. *Mol. Phylogen. Evol.* **54**, 336-343.

3. Beshera, K. A., & Harris, P. M. (2014). Mitochondrial DNA phylogeography of the *Labeobarbus intermedius* complex (Pisces, Cyprinidae) from Ethiopia. *J. Fish Biol.* **85**, 228-245.

4. Durand, J. D., Tsigenopoulos, C. S., Ünlü, E., & Berrebi, P. (2002). Phylogeny and biogeography of the family Cyprinidae in the Middle East inferred from cytochrome *b* DNA—evolutionary significance of this region. *Mol. Phylogen. Evol.* **22**, 91-100.

5. Machordom, A., & Doadrio, I. (2001). Evolutionary history and speciation modes in the cyprinid genus *Barbus*. *Proc. Royal Soc. Lond. B: Biol. Sci.* **268**, 1297-1306.

6. Borkenhagen, K. (2014). A new genus and species of cyprinid fish (Actinopterygii, Cyprinidae) from the Arabian Peninsula, and its phylogenetic and zoogeographic affinities. *Env. Biol. Fish.* **97**, 1179-1195.

7. Tsigenopoulos, C. S., & Berrebi, P. (2000). Molecular phylogeny of North Mediterranean freshwater barbs (genus *Barbus*: Cyprinidae) inferred from cytochrome *b* sequences: biogeographic and systematic implications. *Mol. Phylogen. Evol.* **14**, 165-179.

8. Tsigenopoulos, C. S., Kasapidis, P., & Berrebi, P. (2010). Phylogenetic relationships of hexaploid large-sized barbs (genus *Labeobarbus*, Cyprinidae) based on mtDNA data. *Mol. Phylogen. Evol.* **56**, 851-856.

9. Miya, M. (2009). Whole mitochondrial genome sequences in Cypriniformes. <https://www.ncbi.nlm.nih.gov/nuccore/AP011195>

Table S3. Sample size of sympatric phenotypes within each radiation for estimation of intra-relationships (Fst-values).

|  | Genale R. | Gojeb R. | Didessa R. | Sore R. |
| --- | --- | --- | --- | --- |
|  |  |  |  |  |
| Generalised | 34 | 35 | 35 | 30 |
| Lipped | 18 | 16 | 6 | 7 |
| Scraper1 | 28 | 28 | * | 7 |
| Scraper2 | 19 | 2 | - | - |
| Piscivorous1 | 29 | 13 | 11 | 4 |
| Piscivorous2 | - | - | 1 | - |
| Piscivorous3 | - | - | 1 | - |
| Short | 20 | - | - | - |

- Scraper1 in the Didessa R. is attributed to highly specialized scraper *L. beso*, which originated earlier all other Ethiopian *Labeobarbus* analyzed and genetically well diverged; hence, it is not considered here for intra-relationships.

Table. S4. F_ST_ differences between sympatric phenotypes within each riverine radiation. Lower diagonal: population pairwise F_ST_ based on pairwise differences; upper diagonal: population pairwise F_ST_ based on haplotype frequencies. Significant values shown in bold (significance level = 0.05)

| ***Didessa***  Non-differentiation: Exact P value = 0.000 ± 0.000 * | | | | | | |
| --- | --- | --- | --- | --- | --- | --- |
|  | gen. | lipped | *L. zaphiri* |  |  |  |
| gen. | - | **0.305** | **0.478** |  |  |  |
| lipped | **0.343**** | - | **0.387** |  |  |  |
| *L. zaphiri* | **0.845** | **0.901** | - |  |  |  |
| ***Gojeb***  Non-differentiation: Exact P value = 0.105 ± 0.003 | | | | | | |
|  | gen. | lipped | scraper1 | scraper2 | large-mouthed |  |
| gen. | - | -0.012 | 0.045 | -0.153 | 0.057 |  |
| lipped | 0.009 | - | -0.026 | -0.057 | **0.157** |  |
| scraper1 | 0.058 | 0.040 | - | 0.066 | **0.230** |  |
| scraper2 | -0.130 | 0.001 | 0.101 | - | -0.328 |  |
| large-mouthed | **0.123** | **0.269** | **0.309** | -0.328 | - |  |
| ***Sore***  Non-differentiation: Exact P value = 0.431 ± 0.003 | | | | | | |
|  | gen. | lipped | scraper | large-mouthed |  |  |
| gen. | - | 0.068 | **0.171** | -0.055 |  |  |
| lipped | 0.040 | - | -0.098 | -0.125 |  |  |
| scraper | 0.183 | -0.098 | - | 0.101 |  |  |
| large-mouthed | -0.125 | -0.125 | 0.101 | - |  |  |
| ***Genale***  Non-differentiation: Exact P value = 0.000 ± 0.000 | | | | | | |
|  | gen. | lipped | short | scraper2 | large- mouthed | scraper1 |
| gen. | - | -0.004 | -0.004 | **0.171** | **0.128** | **0.017** |
| lipped | -0.014 | - | -0.003 | **0.173** | **0.128** | **0.011** |
| short | -0.005 | -0.013 | - | **0.182** | **0.134** | **0.015** |
| scraper2 | **0.739** | **0.748** | **0.777** | - | **0.290** | **0.174** |
| large- mouthed | **0.572** | **0.598** | **0.653** | **0.934** | - | **0.117** |
| scraper1 | **0.618** | **0.597** | **0.613** | **0.374** | **0.775** | - |

* Exact test of sample differentiation based on haplotype frequencies - global test of differentiation among sample

** Significantly different pairwise comparisons in exact test of sample differentiation are underscored
